# The Influence of (5′*R*)- and (5′*S*)-5′,8-Cyclo-2′-Deoxyadenosine on UDG and hAPE1 Activity. Tandem Lesions are the Base Excision Repair System’s Nightmare

**DOI:** 10.3390/cells8111303

**Published:** 2019-10-23

**Authors:** Bolesław T. Karwowski

**Affiliations:** DNA Damage Laboratory of the Food Science Department, Faculty of Pharmacy, Medical University of Lodz, ul. Muszynskiego 1, 90-151 Lodz, Poland; Boleslaw.Karwowski@umed.lodz.pl

**Keywords:** (5′*R*)- and (5′*S*)-5′,8-cyclo-2′-deoxyadenisie, tandem lesions, base excision repair, uracil-DNA glycosylase, human AP-site endonuclease 1

## Abstract

DNA lesions are formed continuously in each living cell as a result of environmental factors, ionisation radiation, metabolic processes, etc. Most lesions are removed from the genome by the base excision repair system (BER). The activation of the BER protein cascade starts with DNA damage recognition by glycosylases. Uracil-DNA glycosylase (UDG) is one of the most evolutionary preserved glycosylases which remove the frequently occurring 2′-deoxyuridine from single (*ss*) and double-stranded (*ds*) oligonucleotides. Conversely, the unique tandem lesions (5′*R*)- and (5′*S*)-5′,8-cyclo-2′-deoxyadenosine (cdA) are not suitable substrates for BER machinery and are released from the genome by the nucleotide excision repair (NER) system. However, the cyclopurines appearing in a clustered DNA damage structure can influence the BER process of other lesions like dU. In this article, UDG inhibition by 5′*S-* and 5′*R*-cdA is shown and discussed in an experimental and theoretical manner. This phenomenon was observed when a tandem lesion appears in single or double-stranded oligonucleotides next to dU, on its 3′-end side. The cdA shift to the 5′-end side of dU in *ss*-DNA stops this effect in both cdA diastereomers. Surprisingly, in the case of *ds*-DNA, 5′*S-*cdA completely blocks uracil excision by UDG. Conversely, 5′*R-*cdA allows glycosylase for uracil removal, but the subsequently formed apurinic/apyrimidinic (AP) site is not suitable for human AP-site endonuclease 1 (hAPE1) activity. In conclusion, the appearance of the discussed tandem lesion in the structure of single or double-stranded DNA can stop the entire base repair process at its beginning, which due to UDG and hAPE1 inhibition can lead to mutagenesis. On the other hand, the presented results can cast some light on the UDG or hAPE1 inhibitors being used as a potential treatment.

## 1. Introduction

**Figure 1:**
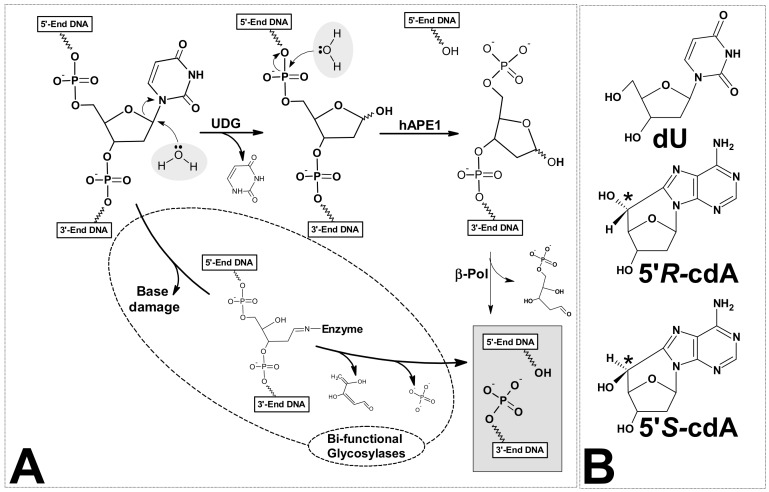
(**A**) Graphical representation of base excision repair system’s (BER) initial step. Difference between mono- and bi-functional glycosylases action; and (**B**) structure of two cdA diastereomes: 5′*R* and 5′*S*.

The genetic information in each cell is written in the sequence of DNA bases. Its stability and reproducibility determine normal cell growth, function and the actual survival of species [1]. In this crucial molecule, DNA lesions are formed continuously as a result of exposure to environmental factors, ionisation radiation, metabolic processes, etc. [2]. Every day, between 10,000 and 1,000,000 DNA incidences of damage per cell are generated as a result of the activity of various factors [3]. That is to say, there are about 3 × 10^17^ natively formed lesions per hour in the whole human body [4]. As a consequence of the variety of lesions, specific nucleic acid repair machineries were developed during cell evolution [5]. However, the effectiveness and reliability of these systems are not 100%. Consequently, changes in a genome can appear, which can subsequently lead to undesirable processes such as carcinogenesis, ageing, etc. [6]. On the other hand, mutation can push evolution forward [7,8]. Moreover, the formation of DNA lesions in privileged sites is the main outcome of much chemotherapy and radiotherapy [9]. It should be pointed out that the structure of DNA damage formed during physiological processes is identical to that formed by external factors [10]. However, the frequency of clustered lesion (CL) formation in native condition is lower than that formed during radiation (Low and High LET radiotherapy) [11]. A clustered lesion formed in both strands of a double helix or a tandem lesion in single-strand are defined as two or more individual lesions within one or two helical turns of the DNA after the passage of a single radiation track [12]. These types of DNA lesions are not only harmful to cells in terms of the repair process (recognition and removal), but also in terms of their accumulation and the fact that they remain in genome for a long time. One hypothesis is that clustered lesions encounter a replication fork in the cell S-phase [13]. It has been found that it takes a cell a good deal longer to repair a cluster composed of dG^oxo^ (7,8-dihydro-8-oxo-2′-deoxyguanosine) and 2-deoxyrionolactone lesions than a solitary lesion; moreover, the mutation frequencies increase dramatically—by up to 58% [14]. In addition, it has been demonstrated that with an increased distance between dG^oxo^, for example, the AP-site (apurinic or apyrimidinic site) and an SSB (single-strand break) in the structure of clustered lesions, the frequency of mutation decreases and the efficiency of the CL repair process increases [15]. In most cases, the presence of dG^oxo^ in clustered lesions is responsible for mutation formation due to GCAT transversion. The majority of DNA damage formed in nuclei or mitochondria, independently of the source of lesion, is removed by base excision repair [16]. Two simple, successive hydrolysis processes start the whole DNA base repair machinery (Figure 1A).

The first reaction is catalysed by specific mono or bifunctional glycosylades [17]. The second is carried out by the apurinic/apyrimidinic site (AP) endonuclease as well as by the bifunctional glycosylase. Human AP-endonuclease-1 (hAPE1) has been found to be the major enzyme which incises a bi-stranded cluster composed of AP-sites [18]. Due to the sensitivity of AP towards environmental changes (temperature, pH, etc.), this molecule could be subject to self-elimination (β- or β,δ-elimination) [19,20]. In 1987, unusual DNA damage was detected in a mammalian cell, namely 5′,8-cyclo-2′-deoxypurines (cdPu). However, historically this type of lesion had been discovered earlier by Keck in 1968 [21,22,23]. This nucleoside modification possesses an additional covalent bond between the C5′ and C8 atoms of the same molecule. Significantly, cdPus are the product of hydroxyl radical action, which initiates a cyclisation reaction by C5′ hydrogen abstraction [24]. Since both moieties (sugar and base) of the nucleoside were modified, cdPus are assigned to a clustered/tandem lesion. The newly formed covalent bond makes the structure of cdPus extremely rigid and insensitive to known glycosylase action. Additionally, they are the pure substrates for polymerase. The 5′*S* diastereomer of 5′,8-cyclo-2′-deoxyadenosine (cdA) is the stopping point for DNA chain elongation by polymerases [25]. It has been also shown that DNA polymerase β (polβ) can efficiently bypass a 5′*R*-cdA, when this lesion is located in complementary strand to the DNA strand under repair but, as can be expected, inefficiently bypass a 5′*S*-cdA [26]. These findings were justified by the previous observation that both diastereomers of cdA located in CAG repeated motive, leads to complementary CTG sequence excision and therefore can be bypassed by polβ in the lagging strand [27]. The influence of 5′,8-cyclo-2′-deoxypurines, in both their diastereomeric forms, on other polymerase activity in humans (polymerase η and ι) and yeast (polymerase ζ), was reported by You et al. [28]. Moreover, RNA polymerase II was the subject of intensive studies toward 5′*S*-cdA overcome during the oligonucleotide transcription process [29].

Neither of the diastereomers of 5′,8-cyclo-2′-deoxypuries (DNA damage) is a substrate for the base excision repair process (BER) and they are both removed from the genome by nucleotide excision repair (NER). Brooks et al. have experimentally shown, using normal and mutant Chinese hamster ovary (CHO) cell extracts isolated from adult hamster brain tissue, that 5′*S*-cdA is repaired by a nucleotide but not the base excision repair system [30].

Additionally, Kuraoka et al. showed that the removal of 5′*S-*cdA and 5′*R-*cdA from DNA is, respectively, 40 and 150 times slower than cis-platin adduct (the chemotherapeutic agent used for the treatment of sarcomas and cancers of the testes, ovaries, bladder, head and neck, and lungs) [31,32]. The above results have been supported by experiments, which have shown that 5′*R* diastereomer of both 5′,8-cyclo-2′-deoxyadenoine/guanosine are better substrates for nucleotide excision repair machinery than their 5′*S* form [33]. It has also been shown that 5′*S*-cdA can influence the dU/AP-site repair process in a typical clustered lesion (bi-stranded). The inhibition of dU repair was observed at a distance of +/− 8 bases [34]. The significance of 5′,8-cyclo-2′-deoxypurines for genome stability and repair processes has been the topic of several review articles [35,36,37].

In this article, the role, influence and differences between both 5′*R* and 5′*S* diastereomers of 5′,8-cyclo-2′-deoxyadenosine (Figure 1B)—denoted as cdA on clustered lesion repair via BER machinery—are shown.

## 2. Materials and Methods

### 2.1. Oligonucleotide Synthesis and Purification

The oligonucleotides, whose sequence is given in Table 1, were synthesized and purified in the Bioorganic Chemistry Department, Polish Academy of Science, Lodz, Poland, on a Geneworld (K & A Laborgeraete GbR, Schaafheim, Germany) synthesizer and using nucleotide phosphoroamidites purchased from the ChemGenes Corporation (Wilmington, MA, USA).

The phosphoroamidite derivatives of (5′*R*)- and (5′*S*)-5′,8-cyclo-2′deoxyadenosine were synthetized as described previously by Romieu et al. [38]. The crude oligonucleotides were purified by HPLC using Varian analytics with UV detection at wavelengths λ = 260nm, Phenomenex (Synergi 4 μm Fusion-RP 80Å, 250 × 4.6 mm) C-18 column.

### 2.2. Oligonucleotide Concentration

The concentration of the obtained oligonucleotides was determined from a maximum of absorbance ~260nm using a Varian Cary 1.3E spectrophotometer (Varian, Brunn am Gebirge, Austria). The online oligonucleotide properties calculator: OligoCalc [39] was used for the extinction coefficient determination of the oligonucleotides.

### 2.3. Mass Spectroscopy of Oligonucleotides

All mass spectra were acquired in the negative-ion mode on a Waters Synapt G2-Si HDMS quadrupole time of flight hybrid mass spectrometer (Waters*,* Manchester*,* UK). Samples of the oligonucleotides were dissolved in 10 mM ammonium acetate with 50% acetonitrile to achieve a concentration of 0.1 OD/mL. Samples were injected into the source of the mass spectrometer using a syringe pump at a flow rate of 10 μL/min. The capillary voltage was set at 2.6 kV, the cone voltage was 40 V, the source temperature was 120 °C, the desolvation temperature was 400 °C, the cone gas was 30 L/h and the desolvation gas was 600 L/h. The data were collected in full-scan negative ion mode over a mass range of 50–2000 *m*/*z*. Data processing was performed with Waters MassLynx 4.1 software (deconvolution with MaxEnt1 function, Waters Corporation, Milfors, MA, USA). The calculated and found masses of oligonucleotides were, respectively, as follows:

12409.14/12409.82 Matrix; 12167.90/12168.25 Cont.dU(0); 12181.98/12182.42 Native; 12165.90/12166.30 ScdA(−7); 12165.90/12166.50 ScdA(−5); 12165.90/12166.46 ScdA(−3); 12165.90/12166.25 ScdA(−1); 12165.90/12166.75 ScdA(+1); 12165.90/12165.20 RcdA(−1); 12165.90/12165.70 RcdA(+1); 12180.90/12181.48 ScdA(+3); 12180.90/12181.54 ScdA(+5); 12180.90/12181.84 ScdA(+7).

### 2.4. Thermal ds-Oligonucleotide Stability—Melting Temperature Measurement

The melting temperatures (Tm) were assigned on a Varian Cary 1.3 E spectrophotometer (Varian) equipped with a multicell block and temperature controller. The required amounts of oligonucleotide strand and complementary strand (0.44 μM and 0.55 μM, respectively) were dissolved in 1 mL of 0.1 M NaCl with 0.01 M MgCl_2_ and hybridized by heating at 90 °C for 10 min followed by slow cooling (overnight). The melting profiles were acquired by measuring the absorbance at a wavelength of 260 nm as a function of temperature. The values, presented in Table 1 as Tm, were measured in a range of between 19 °C and 90 °C with 1.0 °C/min step and one minute of holding time.

### 2.5. Circular Dichroism Analysis of Double-Stranded Oligonucleotides

CD spectra were recorded with a Jasco J-815 dichrograph (JASCO, Easton, MD, USA) at room temperature using 0.1 cm quartz cuvettes and an oligonucleotide concentration of 0.5 μM, as this concentration permitted parallel UV measurements. The CD spectra were collected within the range of 200 to 400 nm with an integration time of 1 s, increment 1 nm, band width 1 nm, scanning speed 50 nm/min. The profiles of the obtained spectra are given in Figure S1.

### 2.6. Preparation of 5′-End-Labeled Oligonucleotides

The oligonucleotides (0.2 μM) were 5′-End-labelled using 3.2 units of T4 polynucleotide kinase (New England BioLabs, Ipswich, MA, USA) with 1.6 mCi (1.6 μL) [γ-^32^P]ATP (Hartman Analytic GmbH, Braunschweig, Germany) in 16 μL of buffer [70 mM Tris-HCl (pH 7.6), 10 mM MgCl_2_, 100 mM KCl, and 1 mM β-2-mercaptoethanol] for 45 min at 37 °C. The protein denaturation was made by sample heating at 95 °C for five minutes. After incubation, the radiolabelled sample was filtrated through a MicroSpin G-25 column (GE Healthcare, Buckinghamshire, UK), at 6000 RPM/2 min, the residue evaporated to dryness. The residue was dissolved in 80 μL of pure H_2_O. The purity of the investigated oligonucleotides was examined on a 20% denaturing polyacrylamide gel.

### 2.7. Oligonucleotide Hybridisation and UDG and hAPE1 Digestion Assay with Subsequent Piperidine Treatment

The labelled oligonucleotide was hybridized, as described previously, with a 1.5-fold excess of the purified non-radiolabelled complementary strand in 80 μL pure H_2_O. UDG and hAPE1 were purchased from NEB (New England BioLabs Ipswich, MA, USA).

The general procedure of UDG and hAPE1 oligonucleotide treatment was as follows: 0.014 μM of oligonucleotides, for each reaction time, presented in Table 1, was dissolved in 5 μL of reaction buffer (pH 7.9 at 25 °C) containing potassium acetate (50 mM), Tris acetate (20 mM), and magnesium acetate (10 mM) DTT (1 mM). To preserve identical experiment conditions for all the presented studies, the amount of digested strand was the same for the single or double-stranded oligonucleotide that was used. The oligonucleotide solution was cooled down in an ice/water bath and the cooled mixture (5 μL) of digestion enzymes UDG and hAPE1 containing 0.6 unit of each was added. (The same buffer as above was used to make the protein solution.) The reactions were incubated at 37 °C for the required time.

After enzymatic treatment, each oligonucleotide sample, if necessary, was precipitated with cold ethanol (100 μL) and 2 μL of glycogen vortex and placed on dry ice for 30 min and subsequently centrifuged at 12,000 rpm for 30 min at 4°C. The ethanol was removed and the residue then dried under reduced pressure at room temperature. If necessary, to reveal the DNA lesions, the dry samples of each investigated oligonucleotide were treated with 100 μL of 1 M piperidine solution at 80 °C for 30 min. The piperidine was then removed under reduced pressure. The residues were dissolved in 7 μL of denaturing loading dye containing bromophenol blue and xylene cyanol, and subjected to electrophoresis on a 20% denaturing polyacrylamide gel containing 7 M urea in 1× TBE for 120 min at a constant power of 44 W. The results of the PAGE electrophoresis analysis were visualized by autoradiography. All the biochemical experiments were repeated three times to confirm that they were reliable and consistent. If necessary, Quantity One 1-D analysis software (Bio-Rad) was used to estimate the cleavage bands.

### 2.8. Theoretical Computation Methodology of ONIOM Studies

As a starting point for the theoretical study, the crystal structure of *ds*-DNA and UDG (1emh.pdb) was chosen [40,41]. Due to the complexity of the system, the following changes were made: the crystal water molecules were removed, the *ds*-DNA was reduced from a ninemer to a pentamer, the protein molecules were restricted only to amino acids from the enzyme active site and next to it. The negative charges of oligonucleotide phosphate groups were neutralized by the addition of protons. This strategy has been well documented as applicable to structural studies of nucleic acids [42,43]. The sequence of the double-stranded pentamer was d[GApUAT]*d[ATATC], the chosen amino acids were Ser273, Leu272 Pro271, Ser270, Pro269, His268, Ala267, Gly246, Ser247, Ser169, Pro168, Pro167, Phe158, Ala214, Gly143, Gl144, Asn204, Asp145, Pro146, Tyr147, His148 (see Figure S2). The protein part was frozen for all studies, leaving the *ds*-DNA part flexible. Due to the cost of the calculation, the ONIOM (Our own N-layered Integrated molecular Orbital and molecular Mechanic) strategy was applied [44,45]. Therefore, the structures of the mentioned systems were divided into high—HL (ds-DNA, B3LYP/6-31G*) and low—LL (protein part B3LYP/3-21G*) levels (layers) of calculation [46,47].

For appropriate nucleoside and hydrogen bond energy calculations, the phosphate or sugar-phosphate backbone was removed from the obtained structures, leaving suitable base pair systems with subsequent atom saturation with the necessary hydrogen atoms. The hydrogen atoms added for saturation were optimised at the B3LYP/6-31G* level of theory, with the position of all other atoms fixed. The optimized structures of the discussed oligonucleotide and UDG (the selected part of enzyme) complexes have been attached as PDB files to the Supplementary Materials, as follows: Cont_pdU.pdb—d[GApUAT]*d[ATATC]//UDG, ScdA(+1)SM.pdb—d[G(5′*S*-cA)pUAT]*d[ATATC]//UDG, ScdA(-1)SM.pdb—d[GApU(5′*S*-cA)T]*d[ATATC]//UDG, RcdA(+1)SM.pdb—d[G(5′*R-*cA)pUAT]*d[ATATC]//UDG and RcdA(-1)SM.pdb—d[GApU(5′*R-*cA)T]*d[ATATC]//UDG. All the calculations were performed in the gaseous phase with the Gaussian 09 (revision A.02, Gaussian Inc., Wallingford, CT, USA) software package [48]. The graphical vitalization of obtained pdf files were made using the Discovery Studio Visualizer v16.1.0.15350 (BIOVIA, San Diego, CA, USA) [49].

## 3. Results and Discussion

To prevent an unexpected mutation, during evolution the cell has developed defence systems which repair and remove effectively, in a reasonable time, a DNA lesion, which is possible mainly due to its “isolated” nature [50]. A good example, which is often used as a single damage model and precursor of AP-site or single-strand break, is 2′-deoxyuridine. This lesion can appear in the nucleus or mitochondrial DNA as a product of direct 2′-deoxycytidine deamination or can be inserted in the genome during polymerise action, etc. However, a clustered lesion is a challenge for the cell repair machinery. The cyclobutane pyrimidine dimer is one of the frequent tandem lesions formed under UV-B/C radiation, which during the repair process can lead to dU formation [51,52].

The commonly accepted nomenclature of the position of mutual lesions in a clustered lesion is as follows: if the DNA damage in one strand is oriented on the 3′-end to the reference lesion in a complementary oligonucleotide, the numbering is positive; if positioned on the 5′-end, then it is negative [53]. In these studies, the same rules have been used to appropriately describe tandem lesions. In terms of damage hierarchy, the dG^oxo^ is repaired after modified pyrimidines like dU, thymine glycol and 5,6-dihydrothymine are removed from DNA [54]. Due to the complexity of CL in comparison to isolated damage, their “lifespan” in the genome is long enough to increase the probability of their appearance in a replication fork during the cell S-phase [55]. Georgakilas et al. have shown that local, multiple damaged sites composed by AP-sites are observed in the cell genome for two weeks. It can be concluded that the slowness of CL repair is the effect of double-strand damage disruption during replication [56]. Therefore, the accumulation of clustered lesions in the genome is significantly dangerous for highly proliferated cells. Additionally, clustered damage can appear in cellular DNA not only as a product of ionisation radiation, but also as a result of endogenous oxidative stress. Oxidative stress is defined as “an imbalance between ROS (reactive oxygen species) production and/or their elimination” [57]. It is important to mention that the amount of DNA damage increases in a cell with weaker repair machinery BER, NER, etc., which is related to the ageing process [58]. Therefore, it can be speculated that the level of CL in cells increases with a longer lifespan [59]. The most crucial point for BER machinery is the modified base recognition and its incision with subsequent AP-site formation (Figure 1A). These two processes can be disrupted by the presence of non-substrate nucleosides such as cdA in a tandem lesion layout [60]. It is important to mention here that no influence of cdA on the dU or AP-site removing from a bi-stranded clustered lesion was denoted [32].

### 3.1. The Influence of 5′R-cdA and 5′S-cdA on UDG and hAPE1 Activity

As a model for this study, dU was chosen as a well-established modification and convenient precursor of an AP-site. The specific glycosylase, UDG (uracil-DNA glycosylase), which is one of the most evolutionary preserved enzymes, removes dU from single and double-stranded DNA (*ss*-DNA, *ds*-DNA) by the pinch-push-plug-pull mechanism [61]. Moreover, dU is removed from *ss*-oligonucleotides three times as fast as from its bi-stranded equivalent [62]. On the other hand, UDG is a monofunctional glycosylase which leaves the linear continuous form of oligonucleotide after glycosidic bond hydrolysis (Figure 1A). Therefore, visualisation of its activity by PAGE radiograms analysis is problematic. Due to the fact that the presence of an AP-site in the genome is highly mutagenic, the influence of cdA on another step in the base repair system was taken into consideration in this study. The most active, common and specific mammalian endonuclease is hAPE1 [63]. As shown in Figure 1A, this enzyme removes the AP-site leaving the 3′ hydroxyl group available for polymerase action. Based on the above, for this study, twelve oligonucleotides with a tandem lesion (dU/cdA) were chosen and used for the investigation of the influence of cyclopurine on dU or AP-site incision, depending on their mutual position. For this purpose, the double and single-stranded forms of DNA were taken into consideration. The DNA sequences are presented in Table 1. The distance between an investigated lesion was changed at an interval of 2 bases, i.e., −/+7, −/+5, −/+3, −/+1. As a control, *ss*- and *ds*-DNA with single dU were used (*ss*-Cont.dU(0), *ds*-Cont.dU(0)).

**Figure 2:**
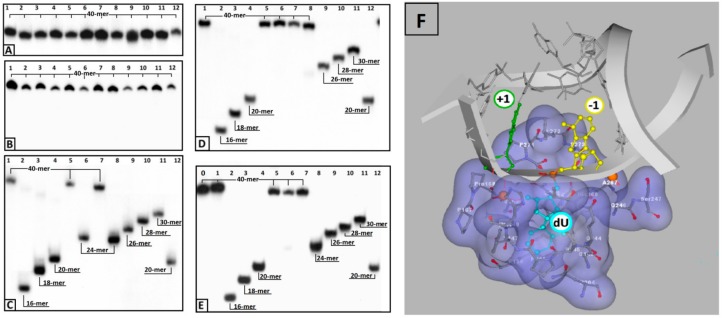
Autoradiograms of denaturing PAGE: (**A**) the purity of *ss*-oligonucleotides; (**B**) *ss*-DNA digested by UDG and hAPE1; (**C**) *ss*-DNA digested by UDG and hAPE1 with subsequent 1M piperidine treatment; (**D**) *ds*-DNA digested by UDG and hAPE1; (**E**) *ds*-DNA digested by UDG and hAPE1 with subsequent 1M piperidine treatment (undigested *ss*-Native oligonucleotide as Line 0). Lines: 1) Native, 2) *S*cdA(-7), 3) *S*cdA(-5), 4) *S*cdA(-3), 5) *S*cdA(-1), 6) *S*cdA (+1), 7) *R*cdA(-1), 8) *R*cdA(+1), 8) *S*cdA(+3), 10) *S*cdA (+5), 11) *S*cdA(+7), 12) Cont.dU(0); (**F**) graphical representation of cdA spatial distribution around the UDG active site (dU) denoted as +1 or −1. The schematic view was built on the basis of a UDG and DNA complex crystal structure denoted as 1emh.pdb [40].

In order to preserve the native conditions as much as possible, UDG and hAPE1 enzymes were used together. The main tenet of this concept was that if one of the proteins was non-active or its activity was disrupted or reduced by cdA, then no shorter oligonucleotide, or indeed, no single-strand break, would be observed. As shown in Figure 2 (Lines 12) in each experiment conditions, the incision of dU and a subsequent AP-site in control single or double-stranded oligonucleotides, *ss*-Cont.dU(0) and *ds*-Cont.dU(0) respectively, were observed, therefore, both enzymes remain active. Moreover, native *ss*-DNA and *ds*-DNA without any modification were not digested by enzymes and were stable during 1M piperidine treatment at 80 °C. Therefore, it can be concluded that the proposed Native oligonucleotide is resistant to each step of the experiments (Figure 2B–D, Lines 1). As mentioned above, the moieties of a clustered lesion (bi-stranded or single-stranded) can influence the mutual repair process, depending on the distances between them. Due to the above, the dU moiety was located at a distance from seven to one base from cdA in both its directions (Table 1). The product, linear continuous strand of lesion digestion by UDG from *ds*- or *ss*-DNA was invisible in PAGE analysis. Consequently, glycosydic bond hydrolysis was carried out in the presence of the AP-site nuclease—hAPE1. This enzyme recognizes the apurinic/apyrimidinic site in a double-stranded oligonucleotide; a single-strand form is not a convenient substrate for its activity [64]. Therefore, no difference in electrophoretic migration between the initial oligonucleotide and digestion product of all of the investigated *ss*-DNA was observed.

**Figure 3:**
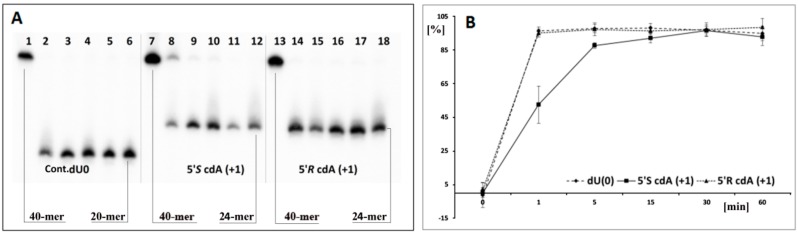
(**A**) Digestion of single-stranded oligonucleotides: Cont.dU(0), *ss*-ScdA(+1) and *ss*-RcdA(+1) by UDG and hAPE1 with subsequent 1 M piperidine treatment. Lines 1–6 correspond to *ss*-Cont.dU(0); Lines 7–12 correspond to *ss*-ScdA(+1); Lines 13–18 correspond to *ss*-RcdA(+1). In all cases, the following order of reaction times was used: 0, 1, 5, 15, 30, 60 min starting from the left site. (**B**) Time scale for dU incision by UDG from *ss*-DNA containing a cluster lesion (The numeric raw data is presented in the graph, and the average and standard deviation values have been given a separate file in the Supplementary Materials). Sequence of single-stranded 40-mer oligonucleotides given in Table 1. (**a**) black points—*ss*-Cont.dU(0), (**b**) black squares—*ss*-ScdA(+1), (**c**) black triangles—*ss*-RcdA(+1).

Based on the above, it can be concluded that cdA does not change the global spatial geometry of a linear oligonucleotide towards the hAPE1 substrate or spontaneous cleavage/rearrangement (Figure 2B). Such a situation forces the use of alternative methods for AP-site incision and single-strand break (SSB) detection. One well-established technique in the field of DNA damage is chemical digestion by 1 M piperidine [65]. The above reaction elucidates that uracil from the investigated clustered lesion (Table 1) is effectively removed by glycosylase from *ss*-oligo, if the distance between cdA and dU is equal to 3 bases or higher in both directions of 5′*S-*cdA. Surprisingly, when dU was shifted to the 5′-site of cdA (*ss*-ScdA(-1) and *ss*-RcdA(-1)), a complete loss of glycosylase activity was noted, irrespective of the cyclopurine diastereomeric forms, 5′*S* or 5′*R* (Figure 2C, Lines 5,7). The situation becomes markedly different when dU was attached to the 3′ hydroxyl group of cdAs. Both oligonucleotides *ss*-ScdA(+1) and *ss*-RcdA(+1) were suitable substrates for UDG. However, the digestion of the glycosidic bond within dU was slower for *ss*-ScdA(+1) than for the control and *ss*-RcdA(+1) oligonucleotides for which the rate of bond cleavage was at the same level (Figure 3). In the same experiment, UDG needs 30 min to achieve the same level of dU removal from *ss*-ScdA(+1) as for the *ss*-Cont.dU(0), and *ss*-RcdA(+1) oligonucleotides. This observation indicates that this type of tandem lesion should be characterised by a prolonged cell lifetime. It should be mentioned here that in some situations, the seemingly easy to remove dU (from the point of privilege flexibility of single-strand oligonucleotides) cannot be rejected from the genome, which subsequently can give rise to increases in mutation or the termination of replication process itself. Kuraoka et al. have shown that a 5′*S-*cdA diastereomer is the end point for known high fidelity polymerases [25].

**Figure 4:**
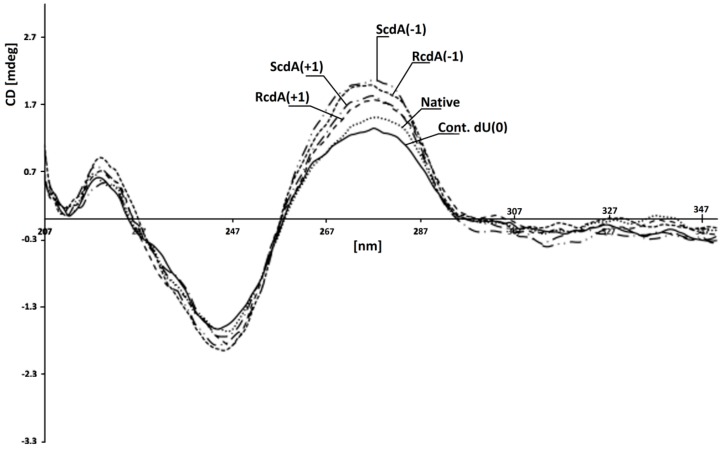
CD spectra of investigated *ds*-oligonucleotides in 0.1 M NaCl with 0.01 M MgCl_2_. Line: *ds-*Cont.dU(0); ••• *ds-*Native; -- *ds-*RcdA(−1); – • – *ds-*ScdA(+1); – •• – *ds-*ScdA(−1); – – *ds-*RcdA(+1). (A large version of Figure 4 as well as separate CD spectra have been added to the Supplementary Materials as a separate file.).

The genetic information is written in the nucleobase sequence and stored in a compressed *ds*-DNA form, i.e., chromatin, in the nucleus. The amount of single-stranded DNA in the cell is rather low [66]. Moreover, most of the enzymes involved in carrying out different processes on DNA recognise and use the double helix as a substrate/template. In the second step of this study, hybridisation of previously investigated *ss*-oligonucleotides with a complementary strand was carried out. This allowed the verification of the influence of the discussed CL on the efficiency of the BER process on the double helix. The melting temperatures (Tm) measurement of *ds*-DNA show their stability in experimental conditions of 37 °C. Table 1 presents the Tm value of all the investigated duplexes [67]. It should be pointed out that the differences in stability between native *ds*-oligo (*ds*-Native) and that with a clustered lesion were in a range of 5 °C. The lowest melting temperature was measured for *ds*-ScdA(+5) at 76 °C. Subsequently, for the native and control *ds*-DNA, Tms were measured at 82 °C and 80 °C, respectively. Despite the differences not being so significant, a tendency could be noted: A) when the dU was placed on the 3′ side of 5′*S*-cdA, the stability of all the investigated oligonucleotides was noted at the same level (79 °C); and B) when dU was shifted to the 5′-end, the Tm value decreases from 80 °C for *ds*-*S*cdA(+1) to 76 °C for *ds*-ScdA(+7), as shown in Table 1. It should be pointed out that the Tm values measured for duplexes containing the opposite 5*’R* diastereomer of cdA, i.e., *ds*-RcdA(+1) and *ds*-RcdA(-1) were equal at 79.02 °C. These observations are in good agreement with previous theoretical studies, which indicated the domino effect in a *ds*-DNA structure forced by 5′*S*-cdA moiety in its 3′-end direction [34]. Conversely, the isomer 5′*R* of cdA left the internal parameters of the double helix as a hydrogen bond or stacking interactions at the same level as in the native B DNA form [68]. Due to the huge problem with 5′*R* diastereomer synthesis of both 5′,8-cyclo-2′-deoxypurines, as well as their insertion within the oligonucleotide, the experimental non-theoretical structural studies of *ds*-DNA (NMR) were made only for their commercially available 5′*S* form [69,70,71]. Fortunately, the crystal structure of (5′*S*)-5′,8-cyclo-adenosine (cA) was achieved, which justified some of the theoretical and biological studies; however, it should be pointed out that even for the rigidity and similarity of cdA and cA in DNA natively, only 5′,8-cyclo-2′-deoxyadenosine is present [72]. However, 5′*R*-cdA forces deformation of the external shape of the double helix spatial geometry similar to that provoked by cis-platin adduct [73]. Surprisingly, the CD spectra of *ds*-oligonucleotides *ds-*ScdA(-1), *ds-*ScdA(+1), *ds-*RcdA(−1), *ds-*RcdA(+1), *ds-*Native and *ds-*Cont.dU(0) showed the same profiles and shifts, in nm, of minimas and maximas: ~245 nm and ~275nm, respectively (Figure 4). The profiles of the obtained spectra are characteristic of the B form of DNA [74,75]. The above results have shown that the investigated oligonucleotides with clustered/tandem lesions can form a stable B-DNA duplex. It should be pointed out that the lack of a methyl group in thymidine (uracil can be perceived as unmethylated thymine) next to cdAs, probably allowed oligo to pack rigid cyclopurine nucleotides in an energetically privileged local double-strand geometry.

**Figure 5:**
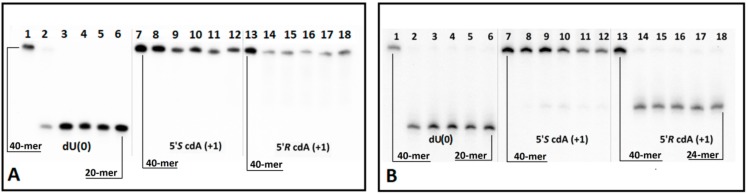
Digestion of oligonucleotides ScdA(+1) and RcdA(+1) in double-stranded forms: (**A**) by UDG and hAPE1 and (**B**) by UDG and hAPE1 with subsequent 1M piperidine treatment. Lines 1–6 correspond to *ds-*Cont.dU(0); lines 7–12 correspond to *ds-*ScdA(+1); lines 13–18 correspond to *ds-*RcdA(+1). In each case, the following order of reaction times was used: 0, 5, 60, 120, 180, 240 min, starting from the left.

The same experiments for *ss*-DNAs were repeated for suitable double-stranded forms. As previously, when the distances between dU and 5′*S*-cdA were higher than one base, both enzymes UDG and hAPE1 exhibited similar activity; as for the control oligonucleotide, *ds*-Cont.dU(0) possessed a sole dU as a reference lesion (Figure 2D). The dU appearing next to the 5′ or 3′ site of 5′*R*- and 5′*S*-cdA did not show in the radiogram image any activity of either one or both of the examined proteins. To elucidate this phenomenon, each reaction mixture was treated by 1 M piperidine. As shown in Figure 2E, for almost all the *ds*-oligonucleotides except one, i.e., *ds*-RcdA(+1), the view was the same as in experiments without piperidine treatment. These results show that clustered lesions dU_PO_5′*S*-cdA and dU_PO_5′*R*-cdA in *ds*-oligo are not a convenient substrate for UDG, as in *ss*-oligo, too. Surprisingly, contrary to previous results, the appearance of 5′*S*-cdA_PO_dU in the double helix, *ds*-ScdA(+1), make the system indigestible or unrecognizable for uracil glycosidase. On the other hand, double-stranded oligonucleotide *ds*-RcdA(+1) (containing 5′*R*-cdA_PO_dU) was recognised and digested by UDG. However, a comparison of the experiment results with and without pyrimidine treatment show that even though a glycosydic bond in dU moiety of CL in *ds*-RcdA(+1) resulted in cleavage by UDG, the subsequently formed AP-site could not be incised by hAPE1 (Figure 2D,E; Lines 8). Moreover, increasing the experiment time by up to four hours did not bring about any benefits, leaving the picture of the radiogram unchanged (Figure 5A,B). The AP-site formed as part of CL in *ds*-RcdA(+1) remained stable. During the same experiment, the single-strand break within the reference oligonucleotide *ds*-Cont.dU(0) was formed within five minutes. Therefore, the appearance of clustered lesion types such as 5′*R*-cdA_PO_dU or 5′*R*-cdA_PO_AP can be highly mutagenic, as they stop hAPE1 activity and leave the undigested apurinic/apyrimidinic site in the genome for a long time. Moreover, due to the reactivity of the aldehyde group of the AP-site, a secondary reaction can take place, leading to an interstrand crosslink formation, for example [76].

### 3.2. Theoretical Study

**Figure 6:**
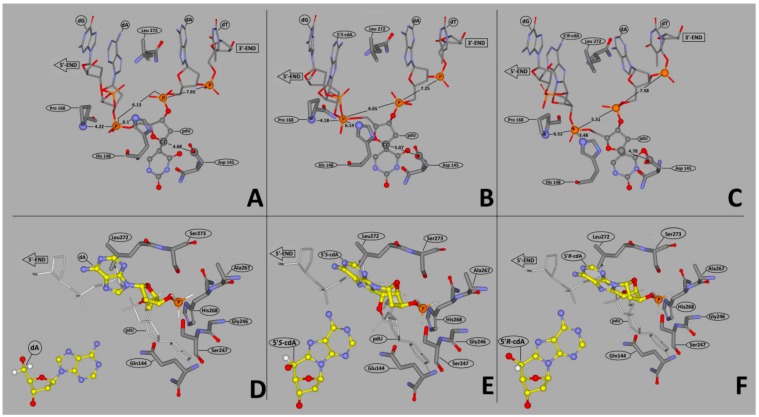
Graphical representation of the distances in Å between the diagnostic amino acids His148/Pro168 and the phosphate group (5′-cdA_PO_pdU-3′), as well as pdU C1’ and His148 endocyclic nitrogen atom: (**A**) *ds-*Cont.pdU, (**B**) *ds-*ScdA(+1) and (**C**) *ds-*RcdA(+1). (**D**) The reference *ds*-oligonucleotide contains 2′-deoxyasdenosine (*ds-*Cont.pdU), (**E**) the oligonucleotide contains (5′*S*)-5′,8-cyclo-2′-deoxyadenosine (*ds-*ScdA(−1)), (**F**) the oligonucleotide contains (5′*R*)-5′,8-cyclo-2′-deoxyadenosine (*ds-*RcdA(−1)) fitting into a suitable protein cave, determined by the amino acids Leu272, Ser273, Ser247, Gly246, Ala267, Gln144, His268.

Glycolytic activity of UDG depends on several structural factors which have been well described in Chattopadhyay’s article [61]. Careful analysis of the UDG and *ds*-DNA crystal structure (1emh.pdb) revealed that a double-stranded oligonucleotide mainly interferes with protein at the distance of three nucleotides (Figure 2F) of a digested strand, whereas the complementary strand interacts only with the intercalating leucine finger (Luciene 272) [40,77]. Due to the fact that the “push” process requires the breaking of hydrogen bonds between dU and the complementary base with subsequent loosening in adjacent base pairs, when hydrolysed by UDG, the 2′-deoxyuridine glycosidic bond is three times as fast in *ss*-oligonucleotide as within a double-stranded one [62]. Contrary to the above, in the single-stranded oligonucleotide, the critical point is only the “plug” stage of the digested machinery. At this point, the enzyme dU or an equivalent, for example pdU (pseudo 2′-deoxyuridine), is inserted into the catalytic pocket [78]. The structural requirements of this process are consistent for the double helix, too. As shown by experimental study after the one base distance crossing, the inhibition effect of cdA on UDG is negligible (Figure 2C–E). Due to the above, in this theoretical study only differences between *ds*-Cont.dU(0), *ds*-ScdA(−1), *ds*-ScdA(+1), *ds*-RcdA(−1) and *ds*-Rcd(+1) were considered. As a starting point, the crystal structure named 1emh.pdb was chosen [40]. 1emh is a complex of UDG and *ds*-oligonucleotide (decamer); moreover, it contains pseudo 2′-deoxyuridine in a glycosylase active pocket. For the geometry optimisation, the ONIOM strategy was used at the B3LYP/6-31G*//B3LYP/3-21G* level of theory. The B3LYP functional was applied due to its established position in the UDG mechanism description [41,79]. Moreover, the double-zeta basis sets (6–31 and 3–21) with a polarisation function were chosen to provide a good electrostatic/stacking interaction as well as a hydrogen bond description [80,81]. On account of the system’s complexity, the protein part was reduced and frozen only to amino acids directly connected or next to the double helix, as shown in the file Cont_pdU.pdb in the Supplementary Materials (Figure 2F). This part (protein) of the molecule was assigned as a low layer of ONIOM and calculated at the B3LYP/3-21G* level of theory. The *ds*-oligonucleotide part was reduced to a pentamer in which the sequence of the nucleobase was converted from d[GTpUAT]*d[ATAAC] to reference *ds*-oligo Cont.pdU: d[GApUAT]*d[ATATC]. The spatial geometry optimisation, at the above level of theory, of the initial complex, without changes, showed only negligible atom displacement. This allowed an investigation of the influence of cdA on pdU “digestion” without any changes in the complex structure, except the position and chirality of cdA, namely the position of the newly formed C5′-C8 covalent bond within cdA. Therefore, the d[GApUAT]*[ATATC], d[G(5′*S*-cA)pUAT]*[ATATC], d[GApU(5′*S*-cA)T]*[ATATC], d[G(5′*R-*cA)pUAT]*[ATATC] and d[GApU(5′*R-*cA)T]*[ATATC] *ds*-oligo can be assigned further as follows: Cont.pdU, ScdA(+1), ScdA(−1), RcdA(+1) and RcdA(−1), respectively. The distance in Å between the 3′ and 5′ phosphate group of pdU for all the investigated structures were as follows: 6.13 Cont.pdU, 6.83 ScdA(+1), 5.31 RcdA(+1), 7.56 ScdA(−1), 6.05 RcdA(−1) (PDB files: Cont_pdUSM, ScdA(+1)SM, ScdA(−1)SM, RcdA(+1)SM, RcdA(−1)SM), as shown in Figure 6A−C. As shown, the distance for RcdA(+1) was the shortest, even shorter than for the Cont.pdU *ds*-oligo; therefore, dU digestion by glycosylase (the pinch stage) should be at least at the same level as for the control *ds*-oligo. Moreover, the pseudo 2′-deoxyuridine in each optimised structure was observed in the catalytic site of UDG. This indicates that if pdU was inserted into the enzyme active pocket, the glycosidic bond should be hydrolysed. The distance in Å between amino acid (carbonyl group) Asparagine 145 (D145) (directly responsible for dU glycosidic bond hydrolysis) and C1’ of pdU was found as follows: 5.07 d[G(5′*S*-cA)pUAT], 4.90 d[GApU(5′*S*-cA)T], 4.78 d[G(5′*R-*cA)pUAT], 4.64 d[GApU(5′*R-*cA)T] and 4.68 d[GApUAT], as shown in Figure 6A–C. This observation—the discussed distance increase from 4.68 Å in d[GApUAT] to 5.07 Å in d[G(5′*S*-cA)pUAT]—allows some basis for an explanation of why dU is not digested in the case of *ds*-ScdA(+1). In this instance, the inhibition of UDG activity by 5′*S*-cdAs, derived from the spatial hindrance forced by the rigid structure of 5′,8-cyclo-2′-deoxyadenosine, makes the *ds*-oligo unpalatable for the pinch-push-plug-pull mechanism. In this case, the hydrogen bond between cdA and thymidine must be traded off to allow the leucine finger to insert dU in the active UDG pocket. Originally, in cdAs, independently of the diastereomeric form 5′*S* or 5′*R*, the 2′-deoxyribose adopted the west (4′O-exo) conformation [82]. Moreover, two-dimensional NOESY NMR studies show that the spatial geometry of (5′*S*)-5′,8-cyclo-2′-deoxyadenosine is stable at a temperature range of between 25 °C and 41 °C [83]. Careful analysis of the investigated complex elucidated that the sugar moiety in 5′*S*-cdA and 5′*R*-cdA adopted the less favourable conformation (3′C-exo) in *ds*-oligo, denoted as *ds*-ScdA(-1) and *ds*-RcdA(-1), as shown in Table 2.

Conversely, when the pdU was attached to the 3′-end of cdA, the 2′-deoxyribose ring in the cyclic nucleoside adopted the privileged conformation 4′O-exo. As shown in Table 2, all the geometrical parameters, i.e., phase, amplitude, puckering and the υ_2_ dihedral angle calculated for the UDG complex with *ds-*ScdA(+1) and *ds-*RcdA(+1), adopted similar values to those obtained for solely nucleosides opposite to constructs possessing *ds-*ScdA(−1) and *ds-*RcdA(−1) oligos [73]. Moreover, the 5′,8-cyclo-2′-deoxyadenosine fitting into a suitable protein cave (determined by the amino acids Leu272, Ser273, Ser247, Gly246, Ala267, Gln144, His268) require energy changes at levels of 15 kcal and 18 kcal in the cases of *ds-*ScdA(−1) and *ds-*RcdA(−1), respectively (Table 3, Figure 6D−F). Contrary to that, the discussed differences were found to be less than 1 kcal and ~4 kcal for *ds-*ScdA(+1), *ds-*RcdA(+1) and Cont.pdU *ds*-oligo respectively (Table 3). The above results obtained for oligonucleotides in which pdU is attached to 5′-end of 5′*S*-cdA or 5′*R*-cdA are in good agreement with previous experimental results.

Additionally, the above observations indicate that in the clustered lesion repair process, the enzyme activity depends on “what*”* is directly attached to the 3′-OH group of the removed DNA damage. These notifications coincide with previous studies about the enzymatic processing of DNA containing tandem dihydrouracil [84]. As has been shown, UDG is less sensitive to the moiety attached to the 5′-end of dU. These phenomena can be explained by the fact that on this site of *ds*-oligo, the structure of the protein cave is much more flexible and malleable, therefore the rigid structure of cdAs can be accommodated. The following distances in Å between diagnostic amino acids His148/Pro168 and the phosphate group (5′-cdA_PO_pdU-3′) of *ds-*ScdA(+1), *ds-*RcdA(+1) and *ds*-Cont.pdU were measured as follows: 6.14/4.18, 3.48/6.32 and 6.1/4.22, respectively (Figure 6A–C). These results did not explain why dU is not digested from *ds*-ScdA(+1), although, based on theoretical studies, it should be. The stability of double-stranded oligonucleotides mainly depends on the stacking interaction and hydrogen bonds formed by complementary nucleobases. Moreover, their energy can allow other molecules, such as intercalators, to penetrate the internal structure of the double helix. The activity of UDG increases as the double helix structure loosens due to a depression of the energy barrier for leucine finger intercalating. The mechanism of the UDG action starts from the “push” point in which the amino acid Leu272 begins to flip out of the dU complementary base and starts to intercalate between the two adjacent base pairs [61]. During this process, the hydrogen bond formed by dU is broken, additionally the adjacent base pairs HB in reference *ds*-oligo (Cont.pdU) become gently debilitated in comparison to the isolated one by 0.9 and 3.76kcal, depending on its position in relation to dU, i.e., +1 or −1 respectively (Table 4).

This clearly shows that UDG is much more sensitive to the changes in the DNA part attached to the 3′-end of dU than to that which is adjacent to its 5′-end. Surprisingly, the energy of the base pair hydrogen bonds formed by 5′,8-cyclo-2′-deoxyadenosine in the protein nucleotide complexes ScdA(+1), ScdA(−1) and RcdA(−1) drastically decreased by up to five kcal. This energy change indicates that pdU, in the above cases, “snuggling down” in the glycosylase active site requires an expenditure of at least 11 kcal. In addition to this value, 17.79 kcal should be added to the U::A HB energy estimated for the isolated base pair. All of this makes the dU recognition process by UDG unprivileged in the light of enzyme fidelity. Therefore, in view of the experiment’s results, in the discussed cases, single-strand breaks were not observed (Figure 2C). The situation is the reverse in the case of *ds-*RcdA(+1) digestion by UDG. The energy change in the cdA::T hydrogen bond adjacent to pdU, which occurred in a hydrolytic cave, registered only a negligible deviation from the value found for the native base pair dA::dT (Table 4), and hypothetically makes the “glycosidic” bond of pdU in *ds-*RcdA(+1) suitable for hydrolysis. These theoretical results are in excellent agreement with data obtained empirically for *ds*-RcdA(+1), as shown in Figure 2E.

## 4. Conclusions

The frequencies of clustered lesions in cell tissue increase as the dose of radiation—Low- or High- LET—increases. This type of genome injury constitutes a significant challenge for the repair systems BER, NER, HEJ, NHEJ, etc. From a molecular pharmacology point of view, during cancer chemotherapy or radiotherapy treatments, such DNA modifications are highly desirable. Therefore, the quest for selective DNA repair protein inhibition is of major scientific interest. In this article, it has been shown that tandem lesion moieties separated by more than one base abolish the inhibition effect of 5′,8-cyclo-2′-deoxyadenosie on UDG, and subsequently on hAPE1 activity. This phenomenon might be derived from the fact that only a small part of oligonucleotide (three nucleotides) crucially and directly interact with the UDG surface, i.e., d[…X_PO_U_PO_X…] in both single and double-stranded DNA forms.

The results of enzymatic digestion of eight *ss*-DNAs and the corresponding *ds*-DNAs, as presented in Table 1, reveal the following:

- the attaching of 5′*S* or 5′*R* cdA to the dU 3′ hydroxyl group in a single-stranded oligo completely blocks UDG activity opposite to the cdA adjacent to the dU 5′-end, allowing UDG glycosidic bond hydrolysis.
- probably due to steric hindrance caused by 5′*S*-cdA in *ss*-ScdA(+1), dU glycosidic bond hydrolysis is slower than that found for *ss*-RcdA(+1) and the reference *ss*-DNA (Cont.dU(0)).
- as shown in Figure 2C,E, both the 5′*S* and 5′*R* diastereomers of cdA inhibit uracil release from the *ds*-RcdA(−1) and *ds*-ScdA(−1) oligos. Surprisingly, only *ds*-RcdA(+1) is a substrate for 2′-deoxyuridine glycosylase and no digestion was observed for *ds*-ScdA(+1). The distance extent between 5′*S*-cdA and dU ranges from −/+3 to −/+7 and probably further base pairs abolished the inhibition effect on glycosylase. Moreover, these tandem lesions were recognised as a good substrate for further AP-site lysis by hAPE1.
- the AP-site formed during *ds*-RcdA(+1) digestion by UDG was identified as an unsuitable substrate for hAPE1.

Finally, it should be pointed out that the appearance of the discussed tandem lesion in the structure of single or double-stranded DNA can stop the entire base repair process at its beginning, which due to hAPE1 inhibition can lead to mutagenesis. On the other hand, these results can cast some light on the UDG or hAPE1 inhibitors being used as a potential treatment.

## Figures and Tables

**Table 1:** Sequence of oligonucleotides containing 2′-deoxyuridine and 5′,8-cyclo-2′-deoxyadenosine and thermal stability (Tm) of corresponding duplexes.

		1	2	3	4	5	6	7	8	9	10	1	2	3	4	5	6	7	8	9	20	1	2	3	4	5	6	7	8	9	30	1	2	3	4	5	6	7	8	9	40	Tm [^o^C]
**Matrix**	**3′**	T	A	G	A	A	C	A	G	T	C	C	T	T	A	T	A	A	C	A	G	A	G	A	T	A	C	G	A	G	G	G	T	G	G	T	T	T	C	C	G	
**ScdA(-7)**	**5′**	C	T	C	T	T	G	T	C	A	G	G	A	A	T	A	T	**U**	G	T	C	T	C	T	**cdA**	T	G	C	T	C	C	C	A	C	C	A	A	A	G	G	C	**79.02**
**ScdA(-5)**	**5′**	C	T	C	T	T	G	T	C	A	G	G	A	A	T	A	T	T	G	**U**	C	T	C	T	**cdA**	T	G	C	T	C	C	C	A	C	C	A	A	A	G	G	C	**79.02**
**ScdA(-3)**	**5′**	C	T	C	T	T	G	T	C	A	G	G	A	A	T	A	T	T	G	T	C	**U**	C	T	**cdA**	T	G	C	T	C	C	C	A	C	C	A	A	A	G	G	C	**79.02**
**ScdA(-1)**	**5′**	C	T	C	T	T	G	T	C	A	G	G	A	A	T	A	T	T	G	T	C	T	C	**U**	**cdA**	T	G	C	T	C	C	C	A	C	C	A	A	A	G	G	C	**79.02**
**RcdA(-1)**	**5′**	C	T	C	T	T	G	T	C	A	G	G	A	A	T	A	T	T	G	T	C	T	C	**U**	**cdA**	T	G	C	T	C	C	C	A	C	C	A	A	A	G	G	C	**79.02**
**ScdA(+1)**	**5′**	C	T	C	T	T	G	T	C	A	G	G	A	A	T	A	T	T	G	T	C	T	C	T	**cdA**	**U**	G	C	T	C	C	C	A	C	C	A	A	A	G	G	C	**80.00**
**RcdA(+1)**	**5′**	C	T	C	T	T	G	T	C	A	G	G	A	A	T	A	T	T	G	T	C	T	C	T	**cdA**	**U**	G	C	T	C	C	C	A	C	C	A	A	A	G	G	C	**79.02**
**ScdA(+3)**	**5′**	C	T	C	T	T	G	T	C	A	G	G	A	A	T	A	T	T	G	T	C	T	C	T	**cdA**	T	G	**U**	T	C	C	C	A	C	C	A	A	A	G	G	C	**77.00**
**ScdA(+5)**	**5′**	C	T	C	T	T	G	T	C	A	G	G	A	A	T	A	T	T	G	T	C	T	C	T	**cdA**	T	G	C	T	**U**	C	C	A	C	C	A	A	A	G	G	C	**76.02**
**ScdA(+7)**	**5′**	C	T	C	T	T	G	T	C	A	G	G	A	A	T	A	T	T	G	T	C	T	C	T	**cdA**	T	G	C	T	C	C	**U**	A	C	C	A	A	A	G	G	C	**76.02**
**Cont.dU(0)**	**5′**	C	T	C	T	T	G	T	C	A	G	G	A	A	T	A	T	T	G	T	C	**U**	C	T	A	T	G	C	T	C	C	**T**	A	C	C	A	A	A	G	G	C	**76.02**
**Native**	**5′**	C	T	C	T	T	G	T	C	A	G	G	A	A	T	A	T	T	G	T	C	**T**	C	T	A	T	G	C	T	C	C	**T**	A	C	C	A	A	A	G	G	C	**82.00**

U = 2′-deoxyuridine; cdA = 5′,8-cyclo-2′-deoxyadenosine; Tm = melting temperature. *A larger version of Table 1 is given in the Supplementary Materials as Table S1 docx file.

**Table 2:** Sugar ring parameters (amplitude, phase puckering and dihedral angle values) of dA relaxed and extracted from optimized structure ds-DNA digested by UDG.

Nucleoside	υ_2_ *	Phase	Amplitude	Conformation
nucleoside relaxed				
dA	−32.67	165.11	33.80	C2′-endo
5′*R*-cdA	10.98	283.23	47.99	O4′-exo
5′*S-*cdA	10.92	283.35	47.31	O4′-exo
nucleoside stressed				
5′*R-*cdA(+1)	−1.07	268.75	48.78	O4′-exo
5′*R*-cdA(−1)	−28.68	236.65	52.18	C3′-exo
5′*S*-cdA(+1)	−2.01	267.58	47.67	O4′-exo
5′*S*-cdA(−1)	−29.63	235.62	52.47	C3′-exo
dA(+1)	−27.20	193.68	27.99	C2′-endo
dA(-1)	−28.79	178.13	28.81	C2′-endo

* dihedral angle υ_2_: C_1’_-C_2′_-C_3′_-C_4′._

**Table 3:**
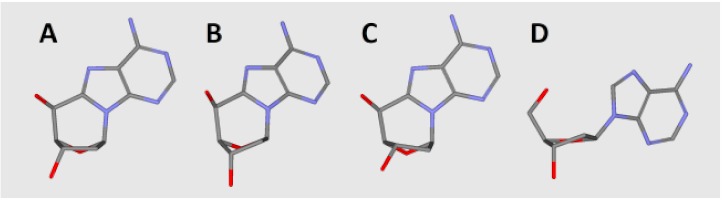
Energy difference in kcal between relaxed nucleosides extracted from *ds-*DNA: 2′-deoxyadenosine, (5′*R*) and (5′*S*)-5′,8-cyclo-2′-deoxyadenosine, calculated at the B3LYP/6-31G ** level of theory. Graphical representation (examples) of 5′,8-cyclo-2′-deoxyadenosine, spatial geometry, extracted from (**A**) *S*cdA(+1), (**B**) *S*cdA(−1) and the relaxed geometry of nucleosides: (**C**) 5′*S*-cdA, (**D**) dA.

Nucleoside	pdU Position +1	pdU Position −1
5′*S*-cdA	−0.61	−15.91
5′*R*-cdA	−0.37	−18.16
dA	−4.62	−4.74

**Table 4:** Next to UDGs’ active site AT base pair hydrogen bond energy, in kcal, presented in Figure 2F, calculated at the B3LYP/6-31G ** level of theory.

cdA Position to pdU	ds-DNA		
	Cont.pdU	*S*cdA	*R*cdA
+1	15.23	5.05	14.88
−1	12.48	5.23	5.23

Unmodified AT base pair: 16.25. Unmodified AU base pair: 17.79.

## Supplementary Materials

The following are available online at https://www.mdpi.com/2073-4409/8/11/1303/s1. Figure S1: CD spectra of investigated *ds*-oligonucleotidestitle, Figure S2: The graphical representation of selected amino acids extracted from 1emh.pdb file, Figure S3: Spectra of Oligonucleotide Mass Spectrometry Analysis, Table S1: Sequence of oligonucleotides, Tables S2: The raw data presented by graphs on Figure.3. Average and corresponding standard deviations values, PDB files: Cont_pdU.pdb, RcdA(+1)SM.pdb, RcdA(-1)SM.pdb, ScdA(+1)SM.pdb, ScdA(-1)SM.pdb and Selected aminoacids indication in 1emh structure.msv.
