# Supplementary material for: The Influence of (5′R)- and (5′S)-5′,8-Cyclo-2′-Deoxyadenosine on UDG and hAPE1 Activity. Tandem Lesions are the Base Excision Repair System’s Nightmare
Source: Cells. 2019 Oct 23;8(11):1303. doi: 10.3390/cells8111303 (PMC6912673; doi:10.3390/cells8111303)

Spectra of Oligonucleotide Mass Spectrometry Analysis

Matrix (mass calculated: 12409.14)

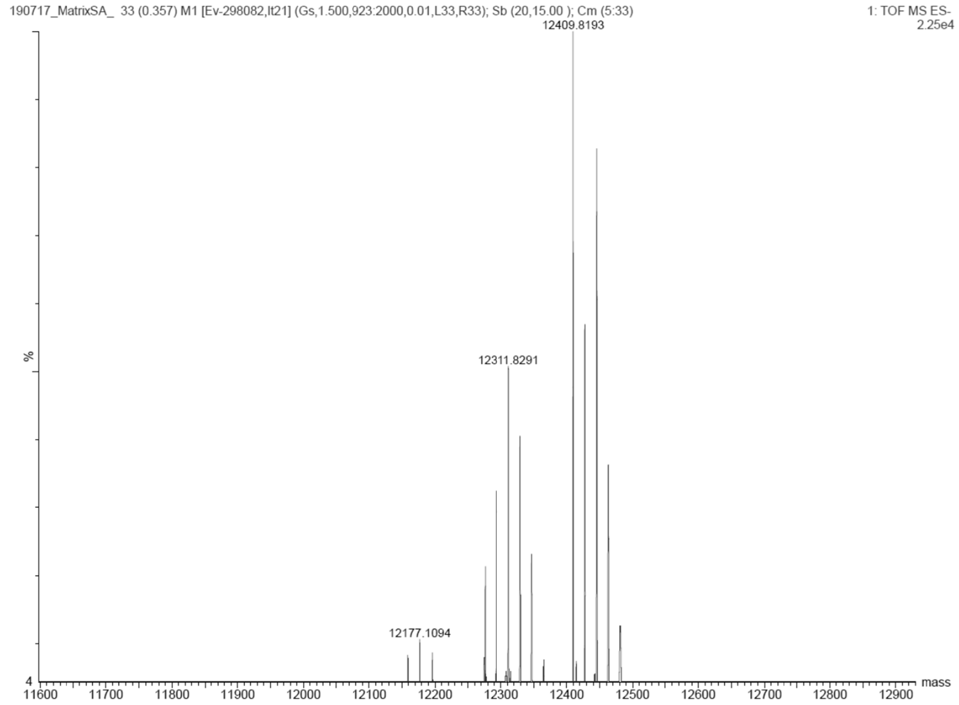

Cont.dU(0) (mass calculated: 12167.90)

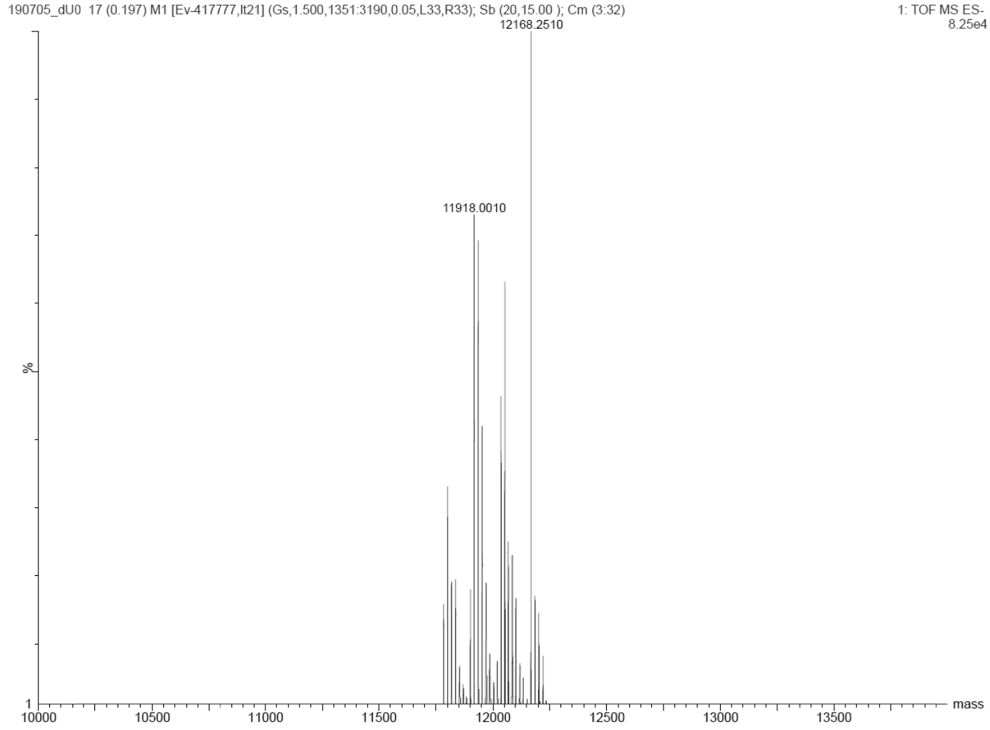

Native (mass calculated:12181.98)

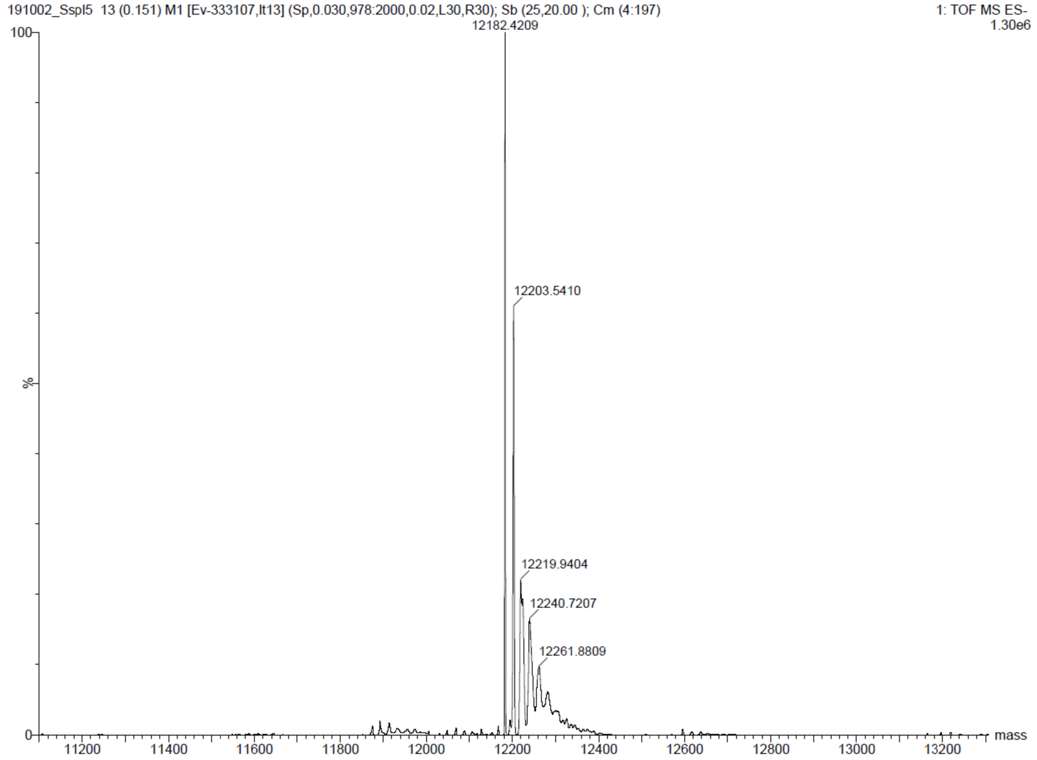

ScdA(-7) (mass calculated: 12165.90)

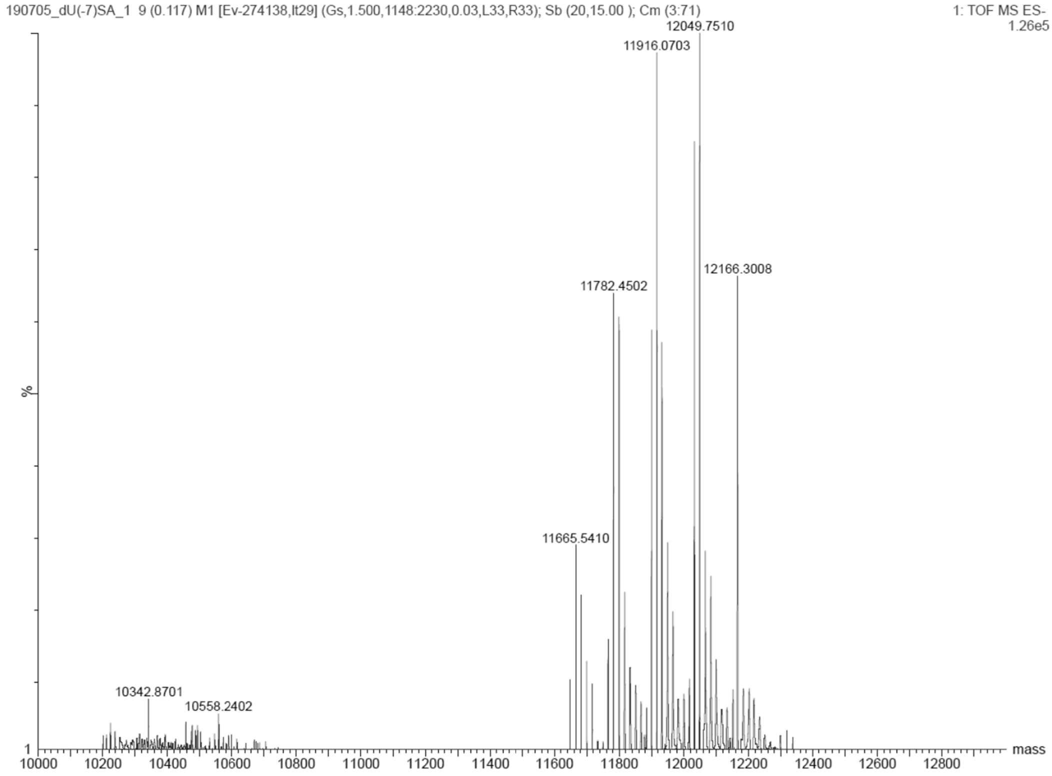

ScdA(-5) (mass calculated: 12165.90)

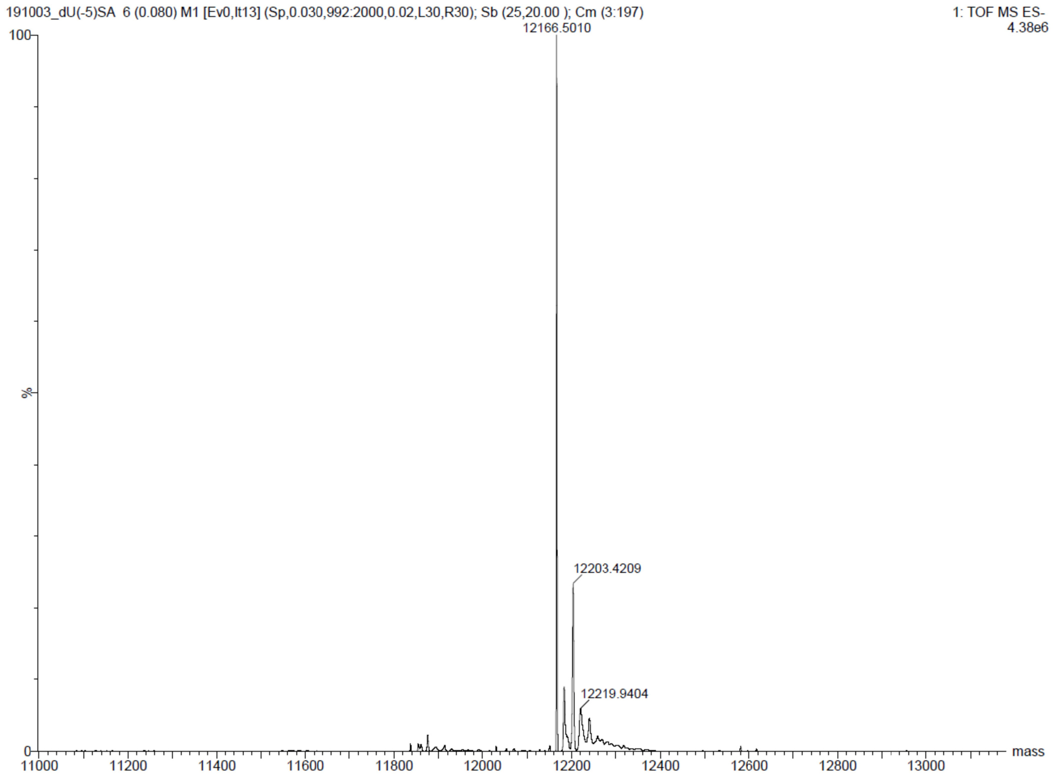

ScdA(-3) (mass calculated: 12165.90)

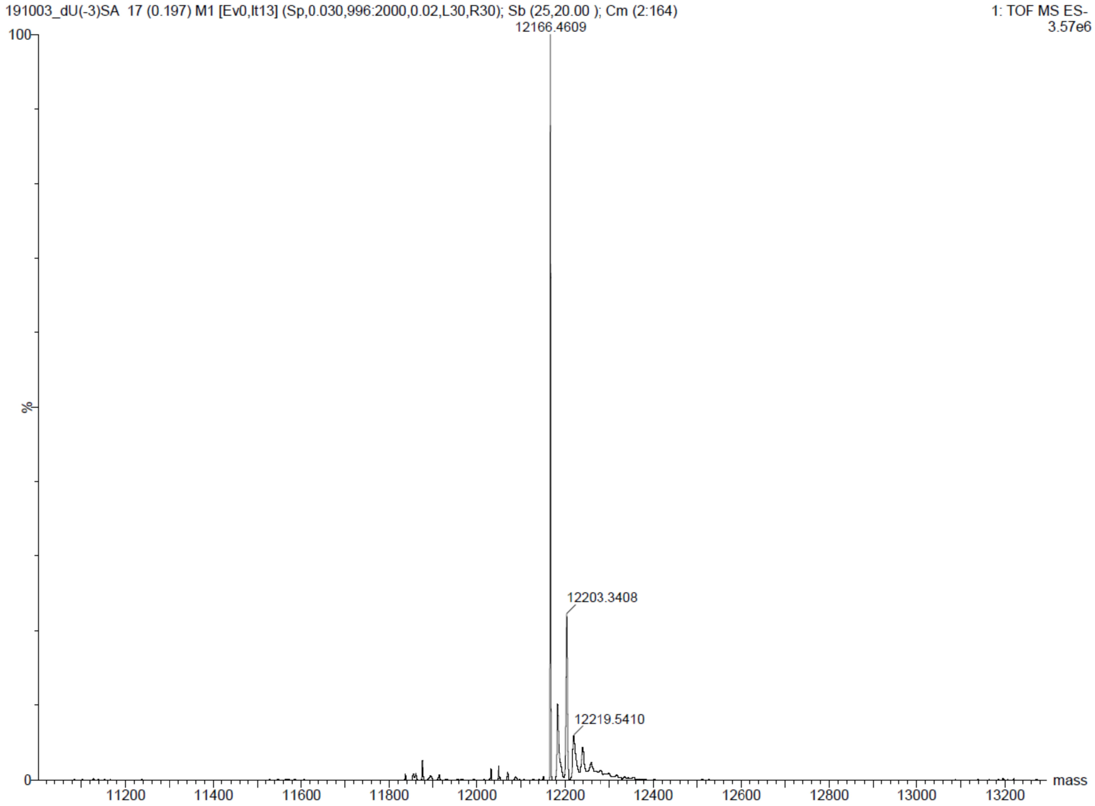

ScdA(-1) (mass calculated: 12165.90)

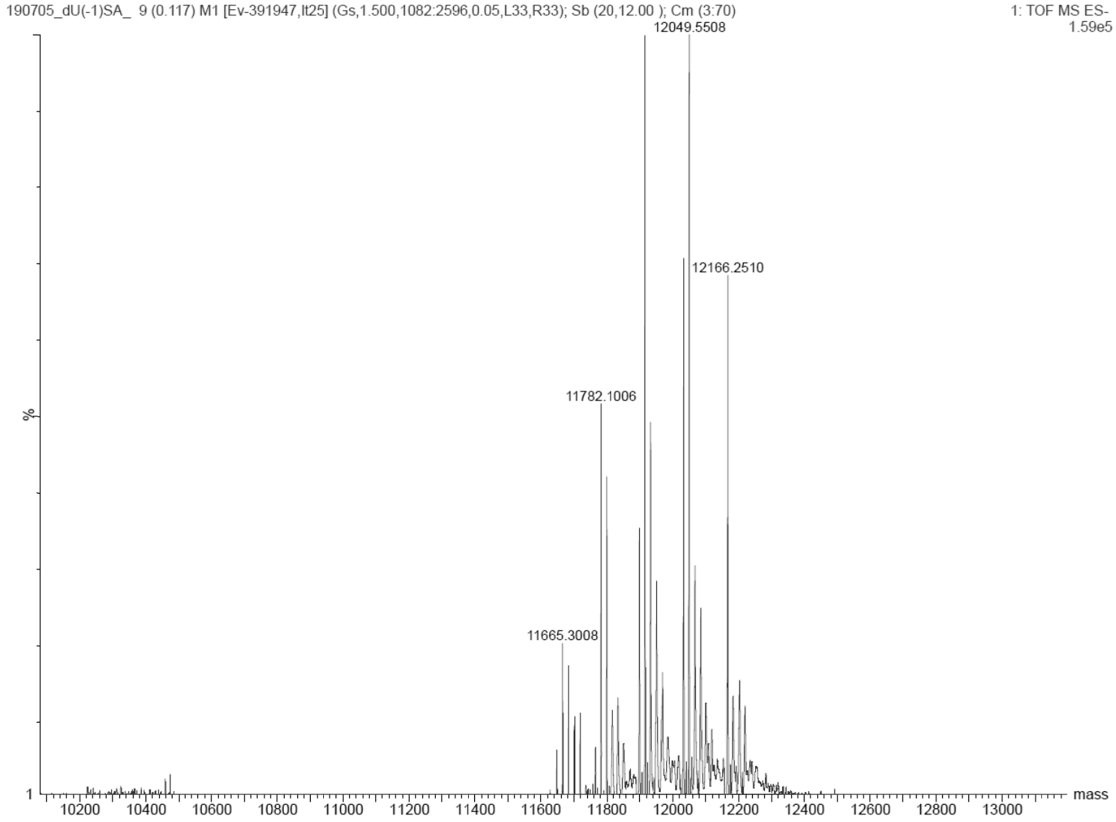

RcdA(-1) (mass calculated: 12165.90)

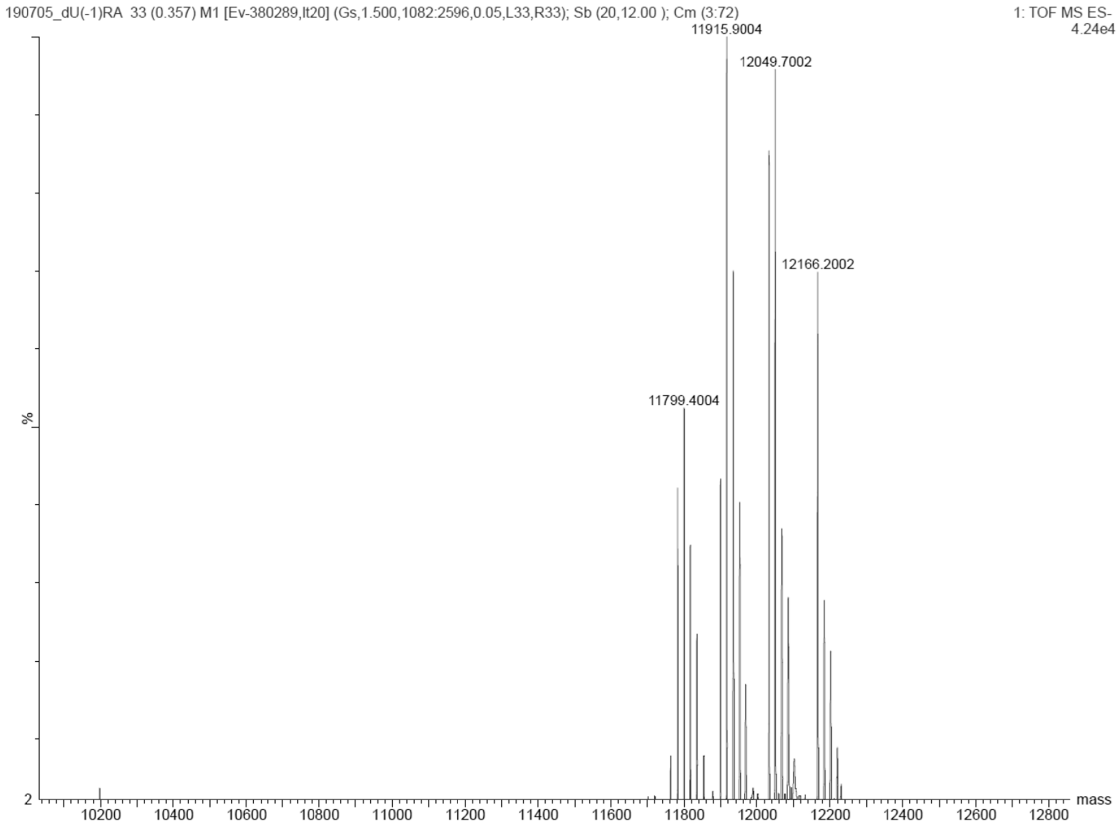

ScdA(+1) (mass calculated: 12165.90)

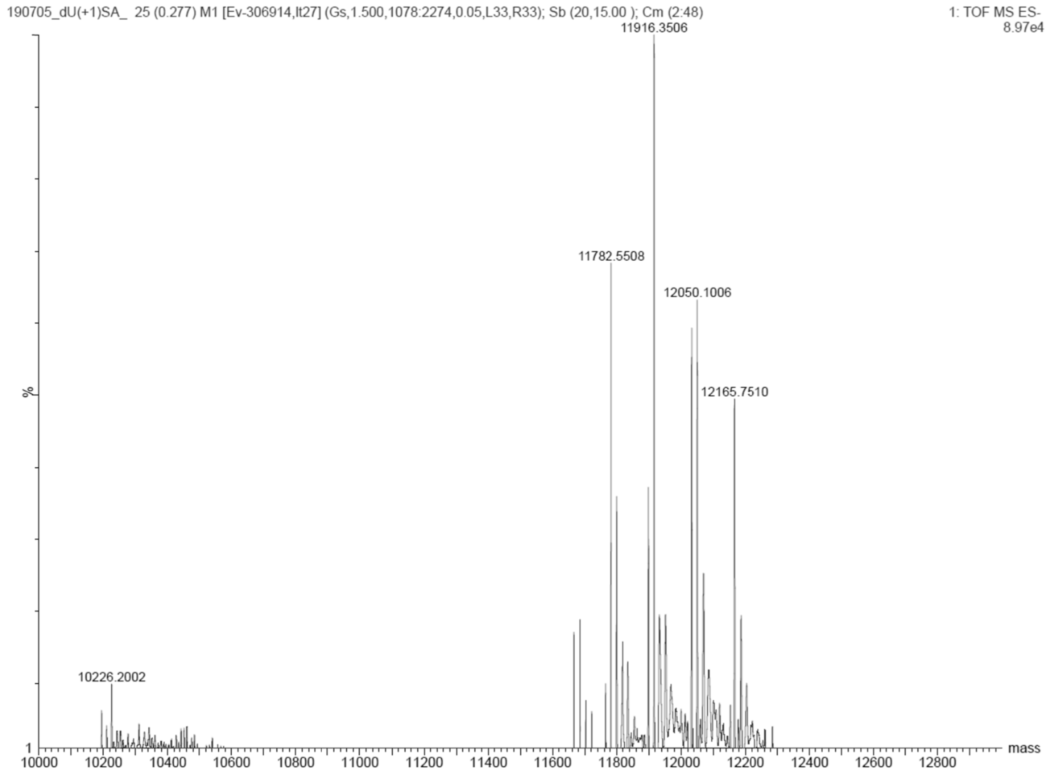

RcdA(+1) (mass calculated: 12165.90)

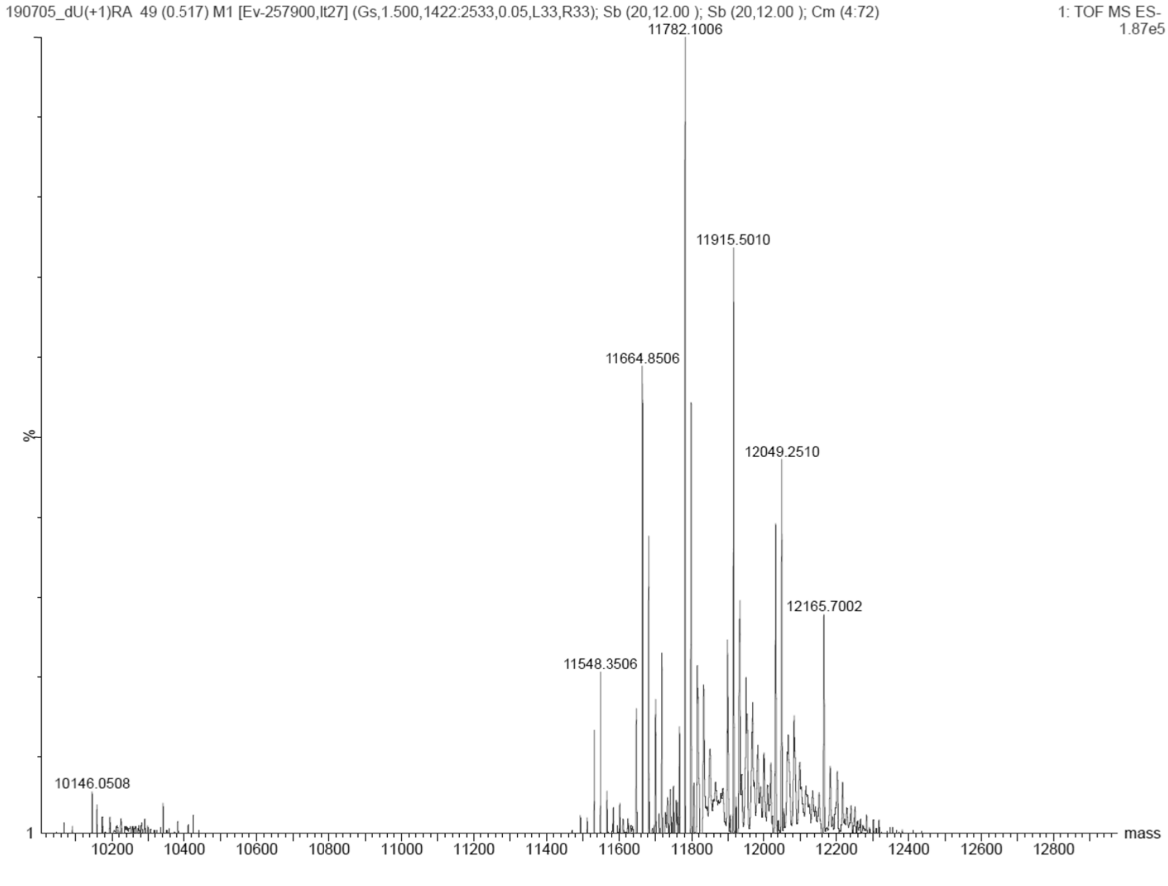

ScdA(+3) (mass calculated: 12180.90)

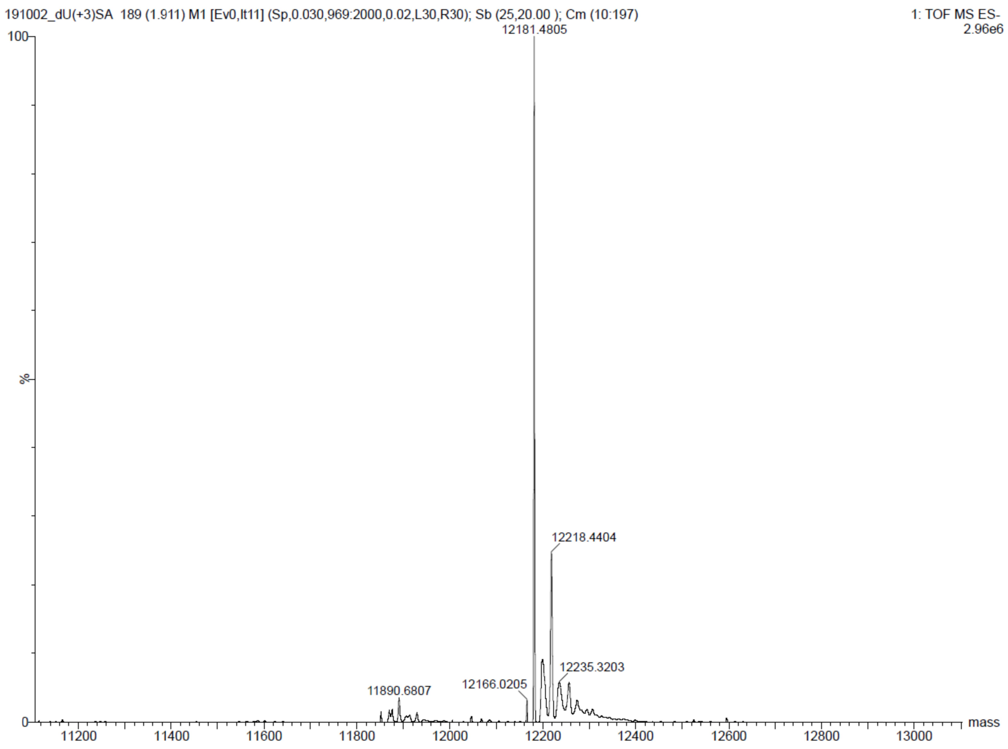

ScdA(+5) (mass calculated: 12180.90)

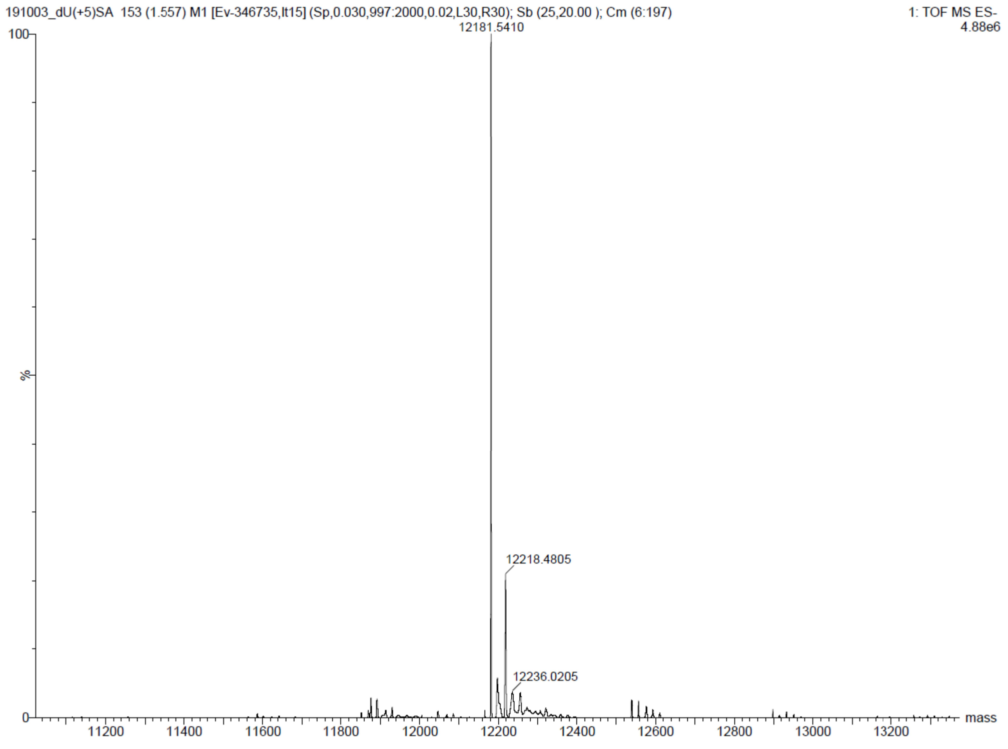

ScdA(+7) (mass calculated: 12180.90)

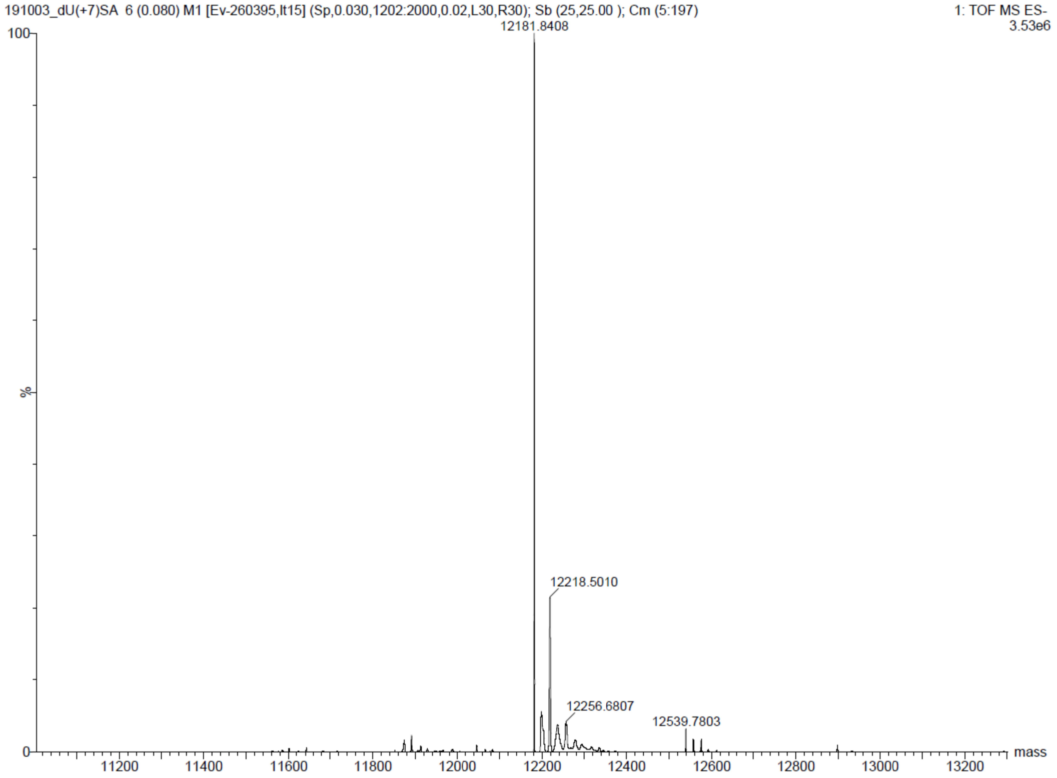

Supplement: Supplementary file 1 [file cells-08-01303-s001.zip › Figure S3.pdf]
