# Supplementary material for: The Influence of (5′R)- and (5′S)-5′,8-Cyclo-2′-Deoxyadenosine on UDG and hAPE1 Activity. Tandem Lesions are the Base Excision Repair System’s Nightmare
Source: Cells. 2019 Oct 23;8(11):1303. doi: 10.3390/cells8111303 (PMC6912673; doi:10.3390/cells8111303)
Supplement: Supplementary file 1 [file cells-08-01303-s001.zip › Tabela S1.docx]

Sequence of oligonucleotides

|  |  | **1** | **2** | **3** | **4** | **5** | **6** | **7** | **8** | **9** | **1**  **0** | **1** | **2** | **3** | **4** | **5** | **6** | **7** | **8** | **9** | **2**  **0** | **1** | **2** | **3** | **4** | **5** | **6** | **7** | **8** | **9** | **3**  **0** | **1** | **2** | **3** | **4** | **5** | **6** | **7** | **8** | **9** | **4**  **0** | **Tm**  **[^o^C]** |
| --- | --- | --- | --- | --- | --- | --- | --- | --- | --- | --- | --- | --- | --- | --- | --- | --- | --- | --- | --- | --- | --- | --- | --- | --- | --- | --- | --- | --- | --- | --- | --- | --- | --- | --- | --- | --- | --- | --- | --- | --- | --- | --- |
| **Matrix** | 3' | **,** | **A** | **G** | **A** | **A** | **C** | **A** | **G** | **T** | **C** | **C** | **T** | **T** | **A** | **T** | **A** | **A** | **C** | **A** | **G** | **A** | **G** | **A** | **T** | **A** | **C** | **G** | **A** | **G** | **G** | **G** | **T** | **G** | **G** | **T** | **T** | **T** | **C** | **C** | **G** |  |
| **ScdA(-7)** | 5' | **C** | **T** | **C** | **T** | **T** | **G** | **T** | **C** | **A** | **G** | **G** | **A** | **A** | **T** | **A** | **T** | **U** | **G** | **T** | **C** | **T** | **C** | **T** | **cdA** | **T** | **G** | **C** | **T** | **C** | **C** | **C** | **A** | **C** | **C** | **A** | **A** | **A** | **G** | **G** | **C** | 79.02 |
| **ScdA(-5)** | 5' | **C** | **T** | **C** | **T** | **T** | **G** | **T** | **C** | **A** | **G** | **G** | **A** | **A** | **T** | **A** | **T** | **T** | **G** | **U** | **C** | **T** | **C** | **T** | **cdA** | **T** | **G** | **C** | **T** | **C** | **C** | **C** | **A** | **C** | **C** | **A** | **A** | **A** | **G** | **G** | **C** | 79.02 |
| **ScdA(-3)** | 5' | **C** | **T** | **C** | **T** | **T** | **G** | **T** | **C** | **A** | **G** | **G** | **A** | **A** | **T** | **A** | **T** | **T** | **G** | **T** | **C** | **U** | **C** | **T** | **cdA** | **T** | **G** | **C** | **T** | **C** | **C** | **C** | **A** | **C** | **C** | **A** | **A** | **A** | **G** | **G** | **C** | 79.02 |
| **ScdA(-1)** | 5' | **C** | **T** | **C** | **T** | **T** | **G** | **T** | **C** | **A** | **G** | **G** | **A** | **A** | **T** | **A** | **T** | **T** | **G** | **T** | **C** | **T** | **C** | **U** | **cdA** | **T** | **G** | **C** | **T** | **C** | **C** | **C** | **A** | **C** | **C** | **A** | **A** | **A** | **G** | **G** | **C** | 79.02 |
| **RcdA(-1)** | 5' | **C** | **T** | **C** | **T** | **T** | **G** | **T** | **C** | **A** | **G** | **G** | **A** | **A** | **T** | **A** | **T** | **T** | **G** | **T** | **C** | **T** | **C** | **U** | **cdA** | **T** | **G** | **C** | **T** | **C** | **C** | **C** | **A** | **C** | **C** | **A** | **A** | **A** | **G** | **G** | **C** | 79.02 |
| **ScdA(+1)** | 5' | **C** | **T** | **C** | **T** | **T** | **G** | **T** | **C** | **A** | **G** | **G** | **A** | **A** | **T** | **A** | **T** | **T** | **G** | **T** | **C** | **T** | **C** | **T** | **cdA** | **U** | **G** | **C** | **T** | **C** | **C** | **C** | **A** | **C** | **C** | **A** | **A** | **A** | **G** | **G** | **C** | 80.00 |
| **RcdA(+1)** | 5' | **C** | **T** | **C** | **T** | **T** | **G** | **T** | **C** | **A** | **G** | **G** | **A** | **A** | **T** | **A** | **T** | **T** | **G** | **T** | **C** | **T** | **C** | **T** | **cdA** | **U** | **G** | **C** | **T** | **C** | **C** | **C** | **A** | **C** | **C** | **A** | **A** | **A** | **G** | **G** | **C** | 79.02 |
| **ScdA(+3)** | 5' | **C** | **T** | **C** | **T** | **T** | **G** | **T** | **C** | **A** | **G** | **G** | **A** | **A** | **T** | **A** | **T** | **T** | **G** | **T** | **C** | **T** | **C** | **T** | **cdA** | **T** | **G** | **U** | **T** | **C** | **C** | **C** | **A** | **C** | **C** | **A** | **A** | **A** | **G** | **G** | **C** | 77.00 |
| **ScdA(+5)** | 5' | **C** | **T** | **C** | **T** | **T** | **G** | **T** | **C** | **A** | **G** | **G** | **A** | **A** | **T** | **A** | **T** | **T** | **G** | **T** | **C** | **T** | **C** | **T** | **cdA** | **T** | **G** | **C** | **T** | **U** | **C** | **C** | **A** | **C** | **C** | **A** | **A** | **A** | **G** | **G** | **C** | 76.02 |
| **ScdA(+7)** | 5' | **C** | **T** | **C** | **T** | **T** | **G** | **T** | **C** | **A** | **G** | **G** | **A** | **A** | **T** | **A** | **T** | **T** | **G** | **T** | **C** | **T** | **C** | **T** | **cdA** | **T** | **G** | **C** | **T** | **C** | **C** | **U** | **A** | **C** | **C** | **A** | **A** | **A** | **G** | **G** | **C** | 76.02 |
| **Cont.dU(0)** | 5' | **C** | **T** | **C** | **T** | **T** | **G** | **T** | **C** | **A** | **G** | **G** | **A** | **A** | **T** | **A** | **T** | **T** | **G** | **T** | **C** | **U** | **C** | **T** | **A** | **T** | **G** | **C** | **T** | **C** | **C** | T | **A** | **C** | **C** | **A** | **A** | **A** | **G** | **G** | **C** | 76.02 |
| **Native** | 5' | **C** | **T** | **C** | **T** | **T** | **G** | **T** | **C** | **A** | **G** | **G** | **A** | **A** | **T** | **A** | **T** | **T** | **G** | **T** | **C** | **T** | **C** | **T** | **A** | **T** | **G** | **C** | **T** | **C** | **C** | T | **A** | **C** | **C** | **A** | **A** | **A** | **G** | **G** | **C** | 82.00 |

U – deoksyurydyna; cdA - 5',8-cyclo-2'-deoxyadenosine; Tm-melting temperature
