# Supplementary material for: The Influence of (5′R)- and (5′S)-5′,8-Cyclo-2′-Deoxyadenosine on UDG and hAPE1 Activity. Tandem Lesions are the Base Excision Repair System’s Nightmare
Source: Cells. 2019 Oct 23;8(11):1303. doi: 10.3390/cells8111303 (PMC6912673; doi:10.3390/cells8111303)
Supplement: Supplementary file 1 [file cells-08-01303-s001.zip › Table S2.pdf]

The raw data presented by graphs on Fig.3. Average and corresponding standard deviations values.

Digestion of single-stranded oligonucleotides: Cont.dU(0), ScdA(+1) and RcdA(+1) by UDG and hAPE1 with subsequent 1M piperidine treatment.

Experiment 1			
	Per cent of Digestion [%]		
Time [min]	Cont.dU(0)	ScdA(+1)	RcdA(+1)
0,0	3,39	2,48	4,92
1,0	93,74	55,63	92,48
5,0	95,10	86,28	93,33
15,0	95,36	88,93	94,22
30,0	94,30	93,12	93,34
60,0	93,44	87,39	93,03

Experiment 2			
	Per cent of Digestion [%]		
Time [min]	Cont.dU(0)	ScdA(+1)	RcdA(+1)
0,0	2,63	1,34	3,76
1,0	96,88	61,66	95,66
5,0	98,50	89,55	97,69
15,0	98,21	94,09	97,08
30,0	98,26	96,68	97,56
60,0	91,51	94,01	99,11

Experiment 3			
	Per cent of Digestion [%]		
Time [min]	Cont.dU(0)	ScdA(+1)	RcdA(+1)
0,0	-9,82	-3,15	-2,23
1,0	98,52	40,24	97,22
5,0	99,78	87,35	100,00
15,0	100,00	93,18	98,25
30,0	98,23	100,00	100,00
60,0	99,78	97,14	100,00

Experiment 1,2,3			
	Average Per cent of Digestion [%]		
Time [min]	Cont.dU(0)	ScdA(+1)	RcdA(+1)
0,0	-1,27	0,23	2,15
1,0	96,38	52,51	95,12
5,0	97,79	87,72	97,29
15,0	98,18	92,06	96,52
30,0	96,93	96,61	97,31
60,0	94,91	92,85	98,52

Experiment 1,2,3			
	Standard Deviation		
Time [min]	Cont.dU(0)	ScdA(+1)	RcdA(+1)
0,0	7,42	2,98	3,84
1,0	2,43	11,05	2,42
5,0	2,42	1,67	3,39
15,0	2,34	2,75	2,07
30,0	2,28	3,44	3,37
60,0	4,33	4,98	3,80
