# Supplementary figures and images for: The Influence of (5′R)- and (5′S)-5′,8-Cyclo-2′-Deoxyadenosine on UDG and hAPE1 Activity. Tandem Lesions are the Base Excision Repair System’s Nightmare
Source: Cells. 2019 Oct 23;8(11):1303. doi: 10.3390/cells8111303 (PMC6912673; doi:10.3390/cells8111303)

CD spectra of investigated *ds*-oligonucleotides

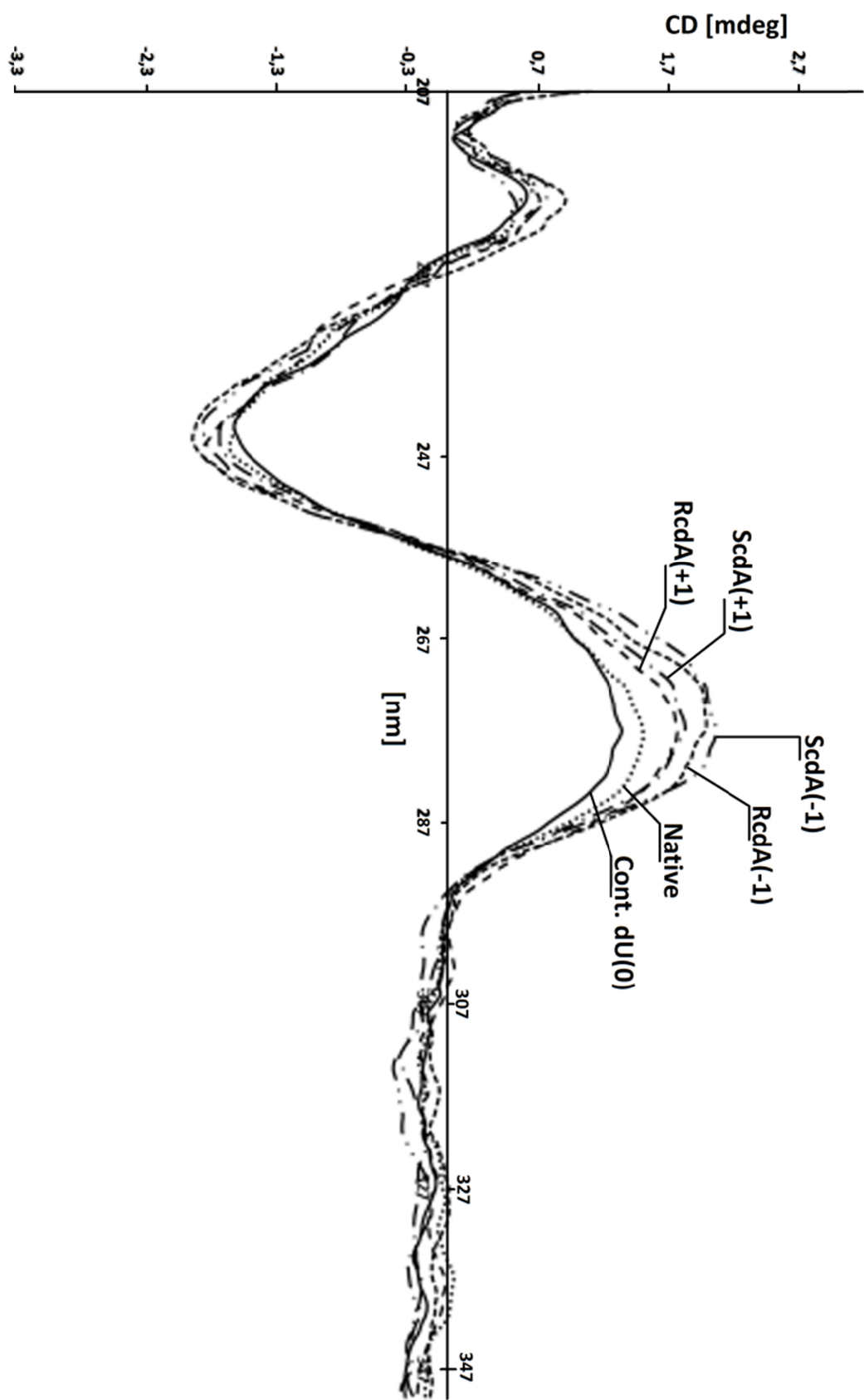

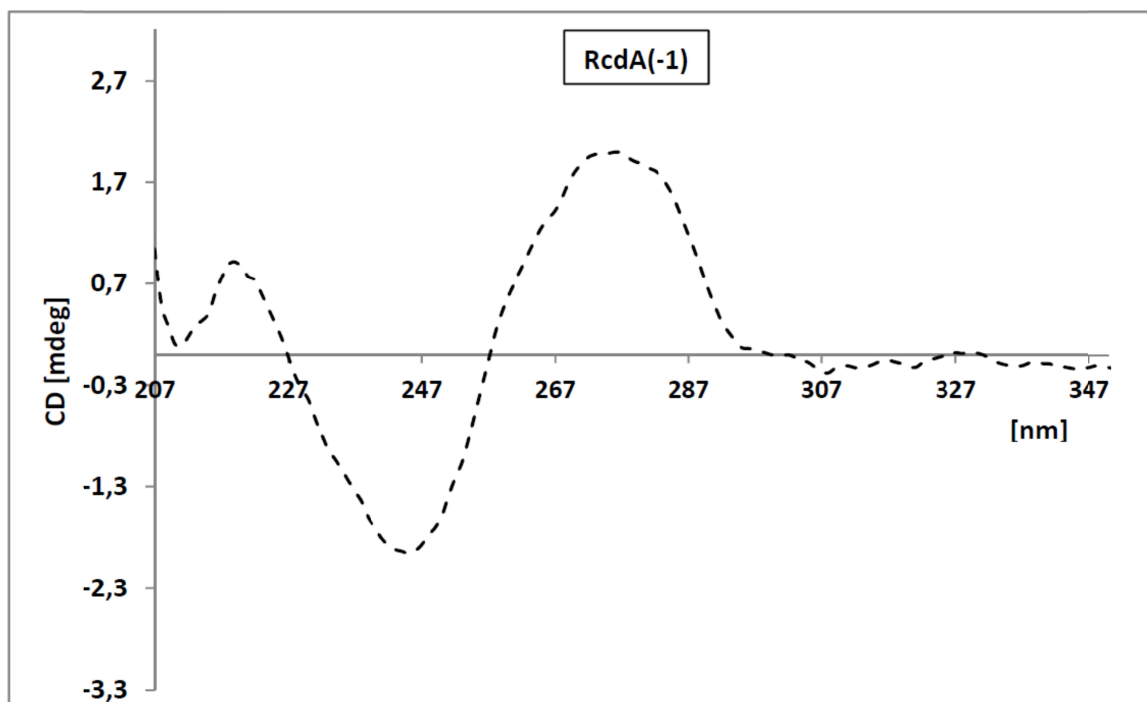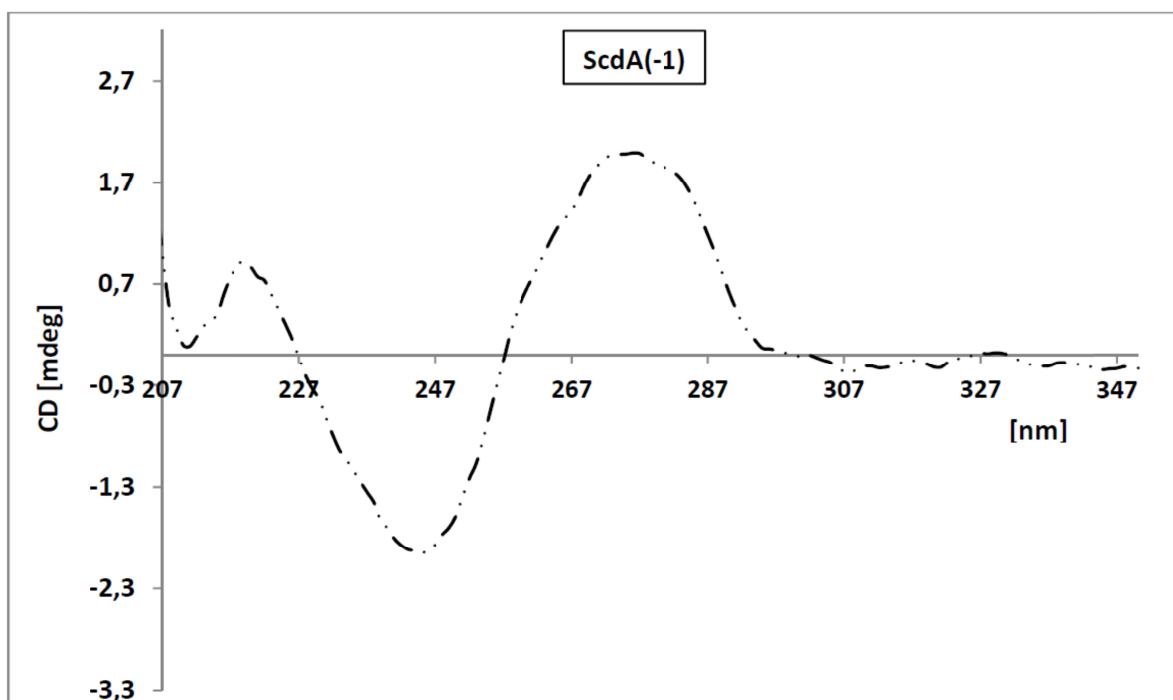

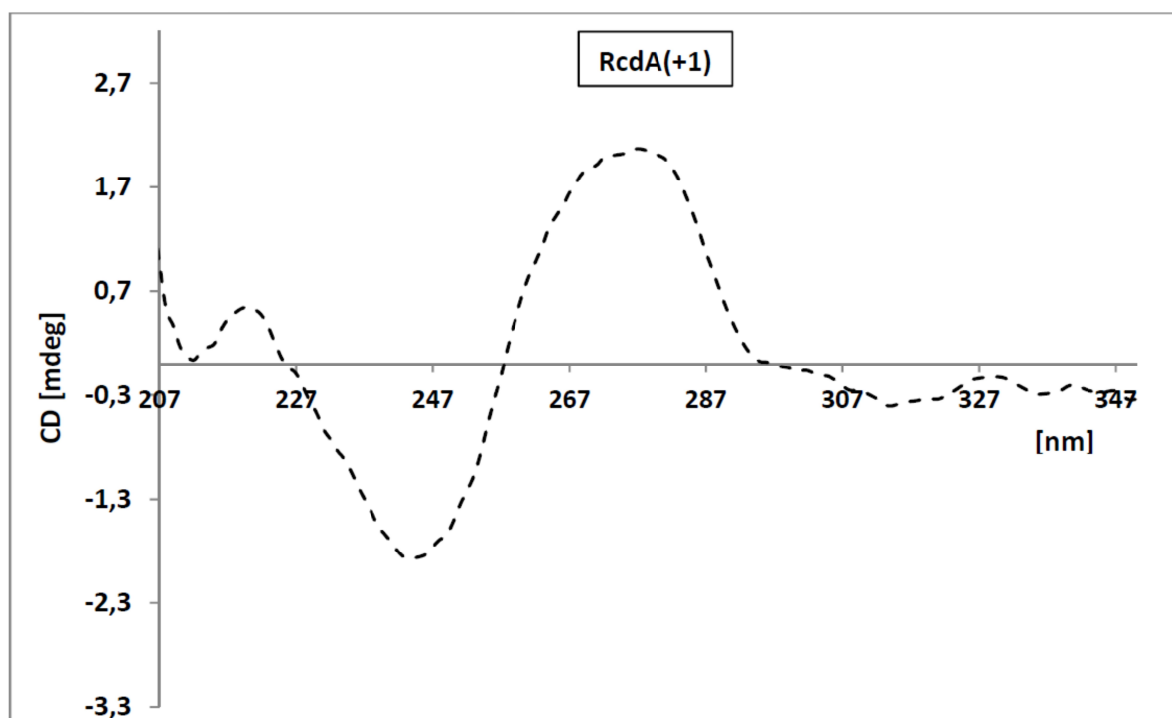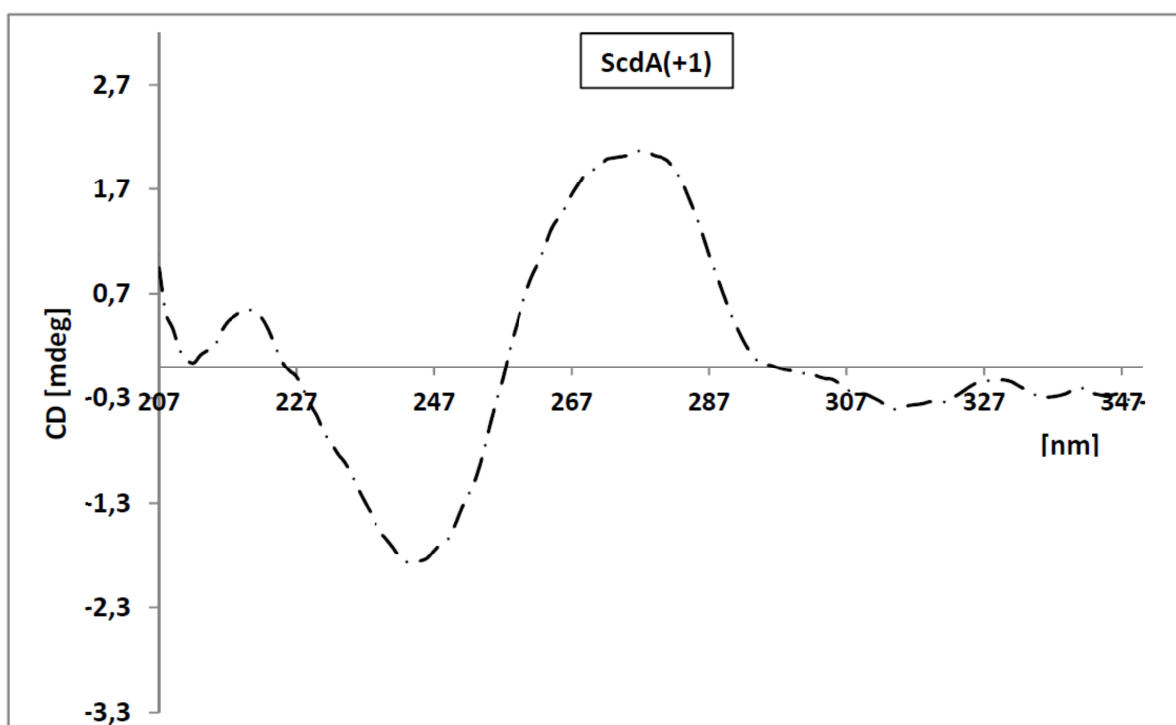

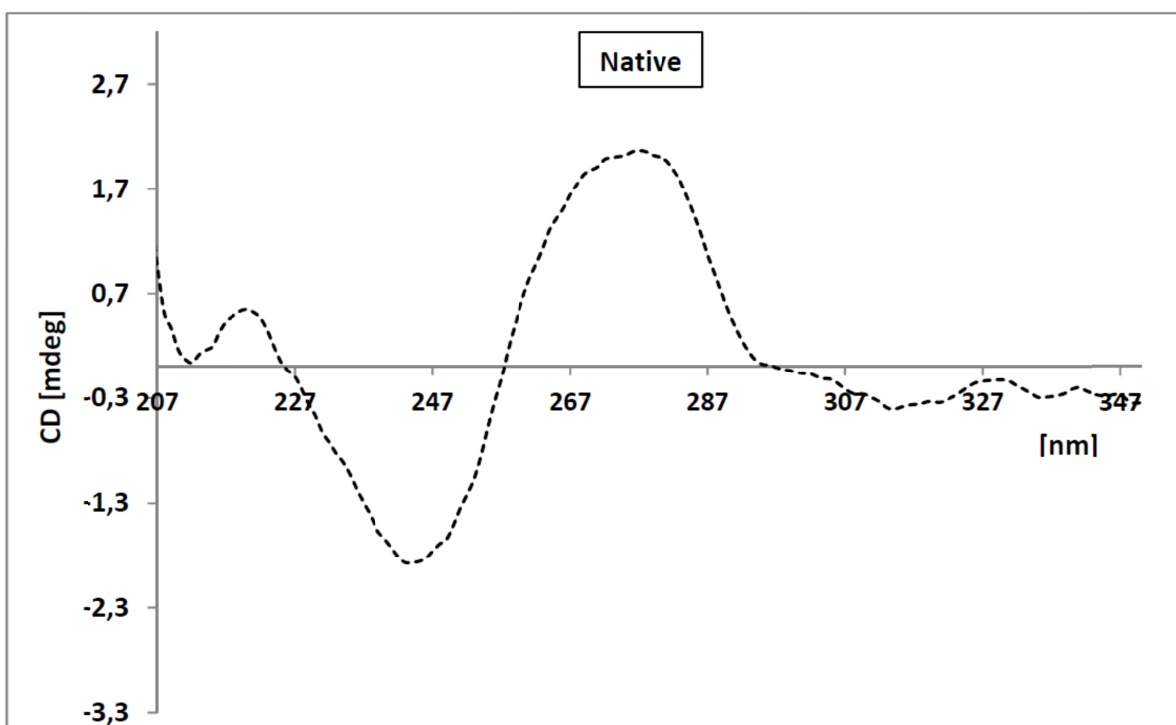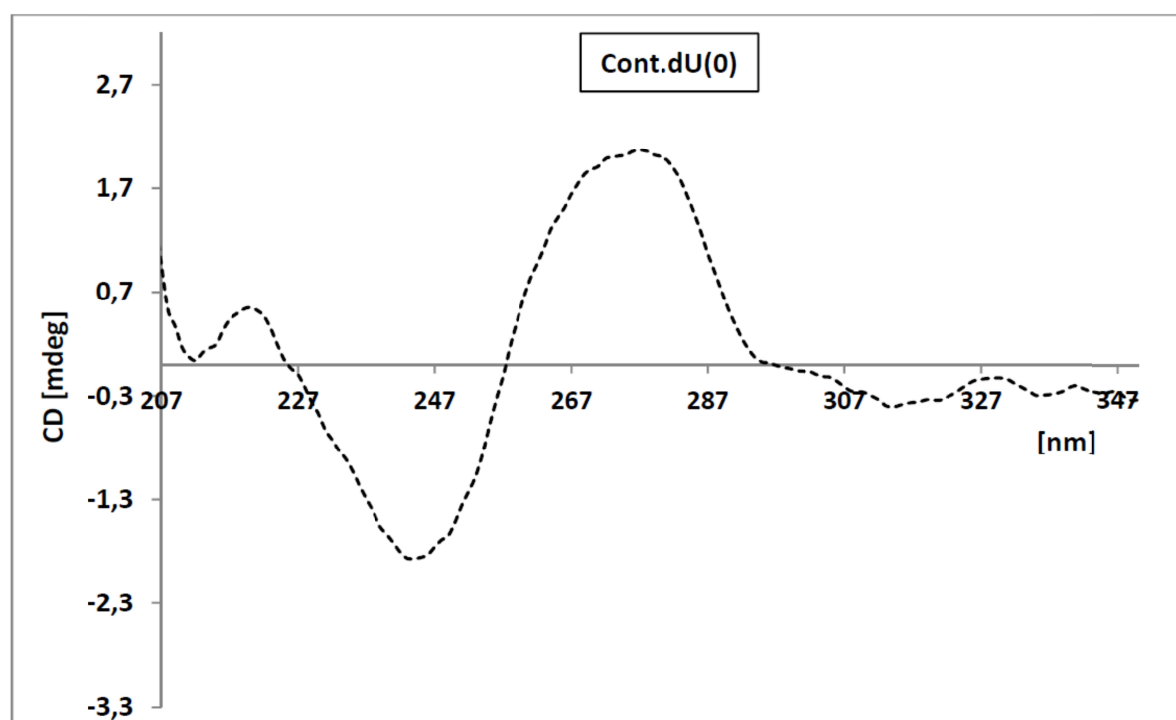

Supplement: Supplementary file 1 [file cells-08-01303-s001.zip › Figure S1.pdf]

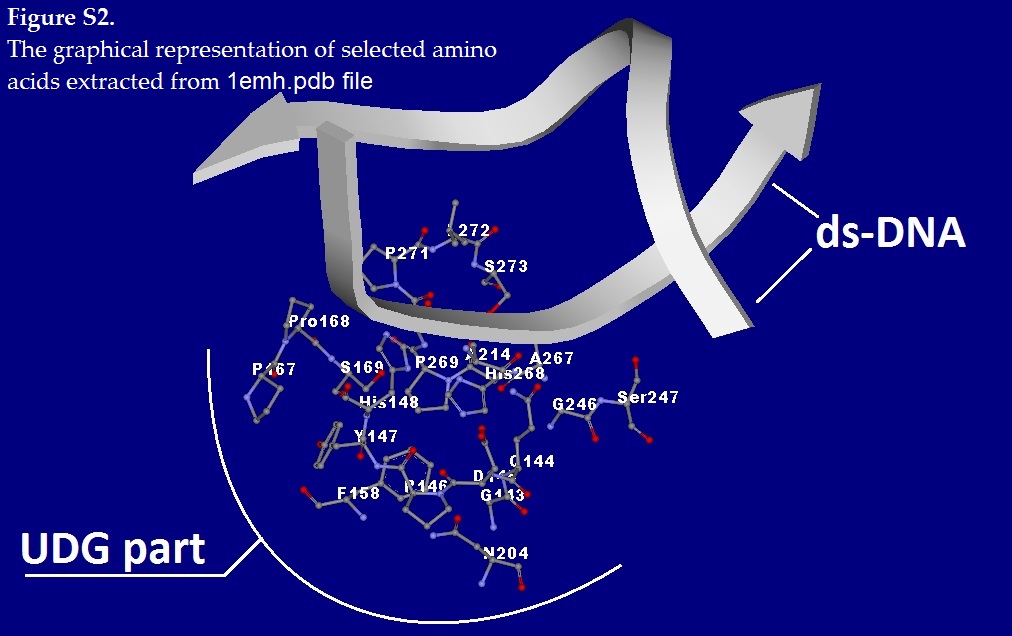

Supplement: Supplementary file 1 [file cells-08-01303-s001.zip › Figure S2.jpg]
